# Understanding PRRSV Infection in Porcine Lung Based on Genome-Wide Transcriptome Response Identified by Deep Sequencing

**DOI:** 10.1371/journal.pone.0011377

**Published:** 2010-06-29

**Authors:** Shuqi Xiao, Jianyu Jia, Delin Mo, Qiwei Wang, Limei Qin, Zuyong He, Xiao Zhao, Yuankai Huang, Anning Li, Jingwei Yu, Yuna Niu, Xiaohong Liu, Yaosheng Chen

**Affiliations:** State Key Laboratory of Biocontrol, School of Life Sciences, Sun Yat-sen University, Guangzhou, Guangdong, China; Institute of Molecular and Cell Biology, Singapore

## Abstract

Porcine reproductive and respiratory syndrome (PRRS) has been one of the most economically important diseases affecting swine industry worldwide and causes great economic losses each year. PRRS virus (PRRSV) replicates mainly in porcine alveolar macrophages (PAMs) and dendritic cells (DCs) and develops persistent infections, antibody-dependent enhancement (ADE), interstitial pneumonia and immunosuppression. But the molecular mechanisms of PRRSV infection still are poorly understood. Here we report on the first genome-wide host transcriptional responses to classical North American type PRRSV (N-PRRSV) strain CH 1a infection using Solexa/Illumina's digital gene expression (DGE) system, a tag-based high-throughput transcriptome sequencing method, and analyse systematically the relationship between pulmonary gene expression profiles after N-PRRSV infection and infection pathology. Our results suggest that N-PRRSV appeared to utilize multiple strategies for its replication and spread in infected pigs, including subverting host innate immune response, inducing an anti-apoptotic and anti-inflammatory state as well as developing ADE. Upregulation expression of virus-induced pro-inflammatory cytokines, chemokines, adhesion molecules and inflammatory enzymes and inflammatory cells, antibodies, complement activation were likely to result in the development of inflammatory responses during N-PRRSV infection processes. N-PRRSV-induced immunosuppression might be mediated by apoptosis of infected cells, which caused depletion of immune cells and induced an anti-inflammatory cytokine response in which they were unable to eradicate the primary infection. Our systems analysis will benefit for better understanding the molecular pathogenesis of N-PRRSV infection, developing novel antiviral therapies and identifying genetic components for swine resistance/susceptibility to PRRS.

## Introduction

Porcine reproductive and respiratory syndrome (PRRS), also called “blue ear” disease due to a typical, but not often observed hallmark of “blue ears”, is widely accepted as being one of the most economically important diseases affecting swine industry. Since its first appearance in the late 1980s in the US and Europe, PRRS has spread worldwide [1], [2], [3]. PRRS is characterized with high mortality in piglets, reproductive failure (late-term abortions and stillbirths, premature farrowing, mummified pigs) in pregnant sows and respiratory disease (interstitial pneumonia, respiratory difficulties) in nursery and grower/finishing pigs, causing highly significant economic losses to the swine industry worldwide, resulting in >$ 560.32 million losses each year in the US alone [4]. The etiologic agent of PRRS is PRRS virus (PRRSV), a small enveloped, linear, single, positive-stranded RNA virus, which is a member of the family Arteriviridae which includes lactate dehydrogenase-elevating virus, equine arteritis virus, and simian hemorrhagic fever virus and enters in the newly established order of the Nidovirales together with the Coronaviridae and Roniviridae family [5]. Since the initial PRRS outbreaks in mainland China in 1996 [2], the PRRSV has spread widely and the infection rate of some swine herds is up to 90% [6], [7]. In June 2006, a so-called “high fever” pig disease large-scalely emerged in China, studies showed that highly virulent Chinese-type PRRSV (H-PRRSV) is the major causative pathogen [8], [9], [10]. However until recently, all PRRSV isolates, including H-PRRSV isolates in China, were identified as the North American type PRRSV (NA PRRSV) genotype [6]. Since 2006, the classical NA PRRSV (N-PRRSV) and H-PRRSV strains coexist in China [6]. PRRSV replicates predominantly in the alveolar macrophage of the lung, can induce prolonged viremia, and cause persistent infections that last for months after initial infection [11]. Infected pigs develop a strong and rapid humoral response but these initial antibodies do not confer protection and can even be harmful by mediating an antibody-dependent enhancement (ADE), since these antibodies can facilitate the entry of the virus into targets cells *in vitro*. In contrast, induction of neutralizing antibodies (NAs) is severedly delayed and their levels remain low, which can not eliminate effectively PRRSV-infected cells [12], [13]. Because of these features of PRRSV infection, PRRS has been one of the most challenging subjects of research in veterinary viral immunology [12].

Regulation of immune responses and genetic resistance to infectious viral diseases is an area of concern for human and swine [14]. PRRSV strongly modulates the host's immune responses, and changes the host's gene expression. Studies showed that PRRSV inhibits type I interferons (IFN-α/β, SPI IFN), especially IFN-α [15], and induces interleukin-10 (IL10) [16], [17]. Because the primary cellular target of PRRSV is the porcine alveolar macrophages (PAMs) of the lung, several studies have analysed the immune responses of PAMs to PRRSV infection. One group [18] used differential display reverse-transcription PCR to identify molecular genetic changes within PRRSV-infected PAMs over a 24 h pi period. Their results suggest that myxovirus resistance 1 (MX1) and ubiquitin specific proteases (USP) genes may play important roles in clinical disease during PRRSV infection. Notably, one recent paper on genome-wide transcriptional response of PAMs following infection with the Lelystad PRRSV strain (European type, EU PRRSV) using Affymetrix microarrays has been published during the preparation of our manuscript [19]. They found that the expression of beta interferon 1 (IFN-β) was strongly upregulated while the expression of IL-10 and TNF-α was weakly upregulated. Almost in the same time, the other group employed Serial Analysis of Gene Expression (SAGE) to examine the global expression of genes in VR-2332 PRRSV strain (North American type, NA PRRSV)–infected PAMs. They identified over 400 unique tags with significantly altered expression levels [20]. *In vitro* studies will be useful for investigating how PRRSV modifies genes expression in primary target cells, such as PAMs. However, many of the outstanding issues will be answered only in the context of PRRSV-infected animals. Hence, the characterization of host immune response under *in vivo* environment to PRRSV is still an area in urgent need of investigation. Lung pathogenesis is a major feature of PRRSV infection. Moreover, in addition to serving as a source of protein in the human diet, the pig is also an excellent biomedical model for humans because of the similarity in size and physiology, and in organ development and disease progression [14]. Thus, understanding the host's immune response to PRRSV infection is important not only for swine production but also for human consumption. However, to date, the immune response to PRRSV in porcine lung has not been analyzed by transcriptome profiling. Next generation high-throughput sequencing technology has been adapted for transcriptome analysis because of the inexpensive production of large volumes of sequence data [21], [22], [23], [24]. The technology developed by Illumina (formerly Solexa sequencing) [25], which is also referred to as Digital Gene Expression (DGE) tag profiling, allows identification of millions of short RNAs in a sample and of differentially expressed genes without the need for prior annotations.

Here we employed the Illumina Genome Analyzer platform to perform a Digital Gene Expression analysis of the porcine lung transcriptome response to N-PRRSV infection, and used histopathology examination to analyze the pulmonary pathological changes of the infected-porcine lungs. The relationship between pulmonary gene expression profiles after N-PRRSV infection and infection pathology was systematically analyzed. The comprehensive analysis of the global host response induced by N-PRRSV suggested an inflammatory response, mediated by multiple inflammatory molecules early during infection that induced tissue injury, an immunosuppressive state, mediated by apoptosis of infected cells, which caused depletion of immune cells and induced an anti-inflammatory cytokine response in which they were unable to eradicate the primary infection. Our systems analysis will benefit for better understanding the molecular pathogenesis of N-PRRSV infection, developing novel antiviral therapies and identifying genetic components for swine resistance/susceptibility to PRRS.

## Materials and Methods

### Ethics Statement

Our study had been approved by Animal Care and Use Committee of Guangdong Province, China. All animal procedures were performed according to guidelines developed by the China Council on Animal Care and protocol approved by Animal Care and Use Committee of Guangdong Province, China.

### Experimental animals and tissue collection

Nine conventionally-reared, healthy 6-week-old, crossbred weaned pigs (Landrace×Yorkshire) were selected from a high-health commercial farm that has historically been free of all major pig diseases, such as PRRSV, porcine circovirus type 2, classical swine fever virus, porcine parvovirus, pseudorabies virus, swine influenza virus and Mycoplasma hyopneumoniae infections. All pigs were PRRSV-seronegative determined by ELISA (HerdChek PRRS 2XR; IDEXX Laboratories) and absence of PRRSV tested by RT-PCR. Pigs were randomly assigned to two groups in the experiment and raised in isolation rooms. Six pigs were inoculated with 6 ml viral suspension (4 ml intranasally and 2 ml intramuscularly) of classical North American type PRRSV (N-PRRSV) strain CH 1a, isolated from China in 1996, gifted by Dr. Zhang Guihong, South China Agricultural University) at a dose of 10^6.0^ TCID50 ml^−1^ on day 0. Three uninfected negative control (UNC) pigs were treated similarly with an identical volume of DMEM culture media from uninfected MARC-145 cells 1 day prior to experimental infection, and were immediately necropsied. N-PRRSV-inoculated pigs were clinically examined daily and rectal body temperatures were recorded from days −2 to 7 pi. Viral re-isolates were performed after the pigs were killed. The infected group showed positive, and the UNC group was negative. Tissue homogenates and serum were examined by N-PRRSV-specific Quantitative PCR (QPCR). The oligonucleotide primers used were NSP2F(5′-GTGGGTCGGCACCAGTT-3′) and NSP2R(5′- GACGCAGACAAATCCAGAGG-3′), designed in the gene segment encoding for NSP2. The TaqMan probe, 5′ FAM-CACAGTTCTACGCGGTGCAGG -TAMRA 3′, was synthesized. Three infected pigs randomly chosen were necropsied at each time point of 96 h pi and 168 h pi. Lung samples were collected from UNC group (C), three pigs at 96 h pi (N96), three pigs at 168 h pi (N168) and immediately frozen in liquid nitrogen for RNA isolation or fixed in 10% neutralized buffered formalin for histological processing.

### Histopathology

Lungs of UNC and experimentally infected pigs were processed routinely for haematoxylin and eosin (H&E) and immunohistochemistry staining, as described previously [26].

### RNA extraction, library construction and sequencing

Total RNA was extracted from frozen lungs using standard protocols (Trizol) and then treated with DNase to remove potential genomic DNA contamination according to the manufactures's protocols. RNA integrity and concentration were evaluated by Agilent 2100 Bioanalyzer (Agilent Technologies).

For RNA library construction and deep sequencing, equal quantities of RNA samples from three UNC individual lungs were pooled, RNA samples from the three infected pig lungs (N96) were pooled, and RNA samples from the three infected individual lungs (N168) were pooled. Approximately 6 µg of RNA representing each group were submitted to Solexa (now Illumina Inc.) for sequencing.

Sequence tag preparation was done with Illumina's Digital Gene Expression Tag Profiling Kit according to the manufacturer's protocol. In brief, mRNA was isolated from 6µg total RNA by binding the mRNA to a magnetic oligo bead. First- and second-strand cDNA were synthesized while the mRNA was attached to the beads. The double stranded cDNAs were digested with NlaIII to wash away all fragmens other than the 3′ CATG fragment attached to the oligo bead. Then GEX NlaIII Adapter 1 was ligated at the site of NlaIII cleavage. In addition, GEX NlaIII Adapter 1 contains the sequence for the restriction enzyme MmeI, subsequently, we applied the restriction enzyme MmeI to create the 17 bp tag. The GEX Adapter 2 was ligated at the site of MmeI cleavage. A PCR with 12 cycles was performed with two primers that anneal to the ends of the adapters to enrich the adapter-ligated cDNA construct. The resulting 85 bp fragments were purified from 6% Novex TBE PAGE gel. Subsequently, the purified cDNA tags were sequenced on the Illumina Cluster Station and Genome Analyzer. Image recognition and base calling were performed using the Illumina Pipeline.

### Analysis of sequencing data

All data is MIAME compliant. The raw data (tag sequences and counts) has been submitted to Gene Expression Omnibus (GEO) under series GSE19970. For the raw data, we filtered adaptor tags, low quality tags and tags of copy number  = 1 to get clean tags. Subsequently, we classified the clean tags according their copy number in the library and show their percentage in the total clean tags and analyzed saturation of the library.

### Tag mapping

The preprocessed database of all possible CATG +17-nt tag sequences was created, using *sus scrofa* UniGene (http://www.ncbi.nlm.nih.gov/UniGene/UGOrg.cgi?TAXID=9823, UniGene Build #35 *Sus scrofa*, Nov, 7th, 2008) from NCBI. For monitoring the mapping events on both strands, both the sense and the complementary antisense sequences were included in the data collection. Information on the position of polyadenylation signals was also collected from the transcript dababase. Then we aligned all clean tags to the reference sequences, and unambiguous tags were annotated. We counted the clean tag number corresponding to each gene.

### Differential expression (DE) detection

To compare the DE of gene across samples (N96/C, N168/C, N168/N96), the number of raw clean tags in each library was normalized to Tags Per Million (TPM) to obtain normalized gene expression level. DE detection of gene or tag across samples was performed according to the previous description [27]. Genes were deemed significantly differentially expressed with a P-value <0.005, a false discovery rate (FDR) <0.01 and an estimated absolute log2-fold change >0.5 in sequence counts across libraries.

### Quantitative PCR (qPCR) analysis

In order to verify the DGE results, we used qPCR analysis. The RNA samples used for the qPCR assays were both the same as for the DGE experiments and independent RNA extractions from biological replicates. qPCRs were done on the Lightcycler480 (Roche), with SYBR-Green detection (SYBR PrimeScript RT-PCR Kit, TaKaRa Biotechnology Co., Ltd.), according to the manufacture's instruction. Each cDNA was analyzed in triplicate, after which the average threshold cycle (Ct) was calculated per sample. The relative expression levels were calculated with the 2^−ΔΔCt^ method. The results were normalized to the expression level of HPRT1 and relative to the C sample.

### Mapping of the DE genes to pig QTLs regions of health traits

Through browsing all health traits of Pig Quantitative Trait Locus (QTL) database (PigQTLdb, http://www.animalgenome.org/QTLdb/pig.html) by trait classes, we obtained mapping details of QTL on the corresponding pig chromosome. Then pig Affymetrix elements corresponding to health trait QTL regions were downloaded to an excel file. By matching the ID of DE genes to all genes in the QTL regions, we obtained DE genes of the corresponding QTL region.

### Pathway analysis of DE genes

Pathway analysis was mainly based on the Kyoto Encyclopedia of Genes and Genomes (KEGG) database. Two-side Fisher's exact test with a multiple testing and χ2 test were used to classify the pathway category. The false discovery rate (FDR) was used to correct the P-value. We chose only pathway categories that had a P<0.05. Within the significant category, the enrichment Re was given by: 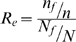 (Re = ENRICHMENT), where n_f_ is the number of flagged proteins within the particular category, n is the total number of proteins within the same category, Nf is the number of flagged proteins in the protein reference database list, N is the total number of proteins in the gene reference database list.

### STC (Series Test of Cluster) and STC-GO (Series Test Cluster of Gene Ontology) analysis

STC is implemented entirely in java. The clustering algorithm first selects a set of distinct and representative temporal expression profiles. These model profiles are selected independent of the data. The clustering algorithm then assigns each gene passing the filtering criteria to the model profile that most closely matches the gene's expression profile as determined by the correlation coefficient. Since the model profiles were selected independent of the data, the algorithm can then determine which profiles have a statistically significant higher numberthan genes assigned using a permutation test. This test determines an assignment of genes to model profiles using a large number of permutations of the time points. It then uses standard hypothesis testing to determine which model profiles have significantly more genes assigned under the true ordering of time points compared to the average number assigned to the model profile in the permutation runs. Significant model profiles can either be analyzed independently, or grouped together based on similarity to form clusters of significant profiles [28], [29].

STC-GO supports Gene Ontology enrichment analyses for sets of genes having the same significant temporal expression pattern. We select random samples of 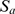 (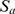 is the number of genes assigned to the same model temporal expression profile r.) genes at each iteration and compute Fisher's exact test p-values for the selected genes in all GO biological categories [30]. The two-sided Fisher's exact test p-value for a category reflects a test of the null hypothesis that the category is enriched in genes assigned to profile r with respect to what would have been expected by chance alone. To decide whether or not to follow up a category that appears enriched in these genes, we would know the statistical reliability of the apparent enrichment. To assess the significance of a particular category, we need to know the distribution of p-values that would occur by random chance. The percentage of false positives to be tolerated will generally depend on the relative costs of false positives and false negatives in whatever follow-up study is to be done. This way of framing the question leads us to specify the false discovery rate (FDR) for a set of categories, rather than significance level (p-value) for each category. With the significance at the 0.05 level, for a given category, the enrichment 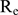 is given by 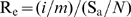 where 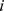 is the number of genes assigned to profile r within the GO category of interest, m is the total number of genes within the GO category of interest, and N is total number of unique genes in the gene reference database list.

## Results

### Clinical and pathologic features of the N-PRRSV-infected pigs

**Figure 1:**
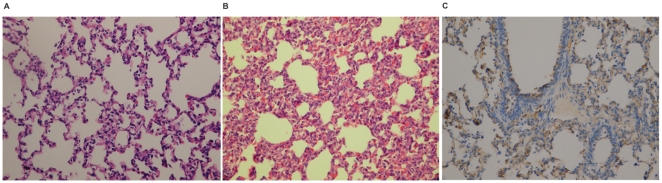
Pathologic examination of lungs infected with the N-PRRSV on day 7 post-infection. (A) Normal morphology observed in UNC porcine lung; (B) Interstitial pneumonia in the lung with thickening of alveolar septa accompanied with infiltration of immune cells; (C) Most viral antigen (brown) was detected in the alveolar cells and the bronchiolar epithelial cells in lesions.

After N-PRRSV infection, the affected pigs exhibited the following clinical symptoms within 3–7 days: fever of 40.08–40.8°C, depression, anorexia, rough hair coats, dyspnoea, reddening of skin, oedema of the eyelids, conjunctivitis, mild diarrhoea, shivering. Those UNC pigs did not show any obvious changes in body temperature and clinical signs. QPCR assay showed that N-PRRSV virus was present in each of the 6 infected pigs. But N-PRRSV NSP2 gene was not differentially expressed at 96 h pi and 168 h pi (Table S1). Histopathology examination of N-PRRSV-affected pigs showed interstitial pneumonia in lungs with thickening of alveolar septa accompanied with infiltration of immune cells (Figure 1B). Most viral antigen was detected in alveolar cells and bronchiolar epithelial cells in lesions (Figure 1C).

### Analysis of DGE libraries

To investigate the regulation of the host response to the N-PRRSV virus, we considered the global gene expression profiles in lungs using Solexa/Illumina's DGE system, a tag-based transcriptome sequencing method. We sequenced three porcine lung DGE libraries from C, N96, N168 using massively parallel sequencing on the Illumina platform. Major characteristics of these three libraries were summarized Table 1. We obtained approximately 6.9 million total sequence tags per library with 515885 distinct tag sequences. Prior to mapping these tag sequences to reference sequences, we filtered adaptor tags, low quality tags and tags of copy number  = 1, producing approximately 6.6 million total clean sequence tags per library with 179589 distinct clean tag sequences. The C library had the highest number of both total sequence tags and distinct sequence tags; this was followed by the N168, N96 libraries. Moreover, the C library had the highest ratio of number of distinct tags to total tags and the lowest percentage of distinct clean high copy number tags. The data showed that more genes were detected in the C library than other two libraries and more transcripts were expressed at lower levels in the C library. Saturation analysis of capacity of libraries showed that new emerging distinct tags were gradually reduced with increasing of total sequence tags when the number of sequencing tags was big enough. When the number of sequencing tags reached 3 million, library capacity approached saturation (Figure S1).

**Table 1:** Major characteristics of DGE libraries and tag mapping to the UniGene transcript database.

	C		N96		N168	
	Distinct Tag	Total Tag	Distinct Tag	Total Tag	Distinct Tag	Total Tag
Raw Data	789417	7767249	377998	6257249	380240	6659396
Low Quality Tag	23718	26271	4775	7247	4443	6698
Adaptor Tag	1	83	1	22	1	30
Tag CopyNum = 1	516291	516291	231292	231292	228364	228364
Clean Tag	249407	7224604	141930	6018688	147432	6424304
CopyNum >1	249407	7224604	141930	6018688	147432	6424304
CopyNum >5	80567	6765151	54053	5776605	56305	6172785
CopyNum >10	50762	6540885	36201	5641671	37717	6032477
CopyNum >20	32580	6276679	24523	5471275	25596	5855124
CopyNum >50	17820	5804733	14472	5146248	14849	5508658
CopyNum >100	10562	5288358	8989	4755293	9125	5099881
Tag Mapping						
All Mapping	118980	5228761	70709	4533457	75250	4921979
Unambiguous Mapping	98305	3961692	58288	3331762	62087	3562350
Unknown Tag	130427	1995843	71221	1485231	72182	1502325

All Mapping represents the number of all tags mapped to the UniGene virtual tag database, Unambiguous Mapping represents the number of unambiguous tags mapped to the UniGene virtual tag database, unambiguous tags indicate the tags matched only to one gene.

### Analysis of tag mapping

For tag mapping, we preprocessed one reference tag database that included 51670 sequences from *sus scrofa* Unigene. To get the reference tags, we used NlaIII to digest all the samples and took all the CATG+17 tags in the gene as the gene's reference tags, not only the 3′ most one. We obtained 194664 total reference tag sequences with 172119 unambigous tag sequences. Considering polymorphism across samples, tolerances were set to allow one mismatch in each alignment. By the criteria, 47.71%∼51.04% of distinct clean tags mapped to the Unigene virtual tag database, 39.42%∼42.11% of the distinct clean tags mapped unambiguously to the Unigene, and 52.29%∼48.96% of the distinct clean tags didn't map to the Unigene virtual tag database (Table 1). The occurrence of unknown tags was probably due to the incompleteness of pig genome sequencing. Most Solexa experimental tags matched to the 1st or 2nd 3′ CATG site in high-confidence transcripts (Figure S2). For solexa sequencing can distinguish between transcripts originating from both DNA strands, employing the strand-specific nature of the sequencing tags obtained, we found evidence for bidirectional transcription in 6368 to 8271 of all detectable Unigen clusters and 3889 to 4043 antisense-stand specific transcripts (Table S2). By comparison, the ratio of sense to antisense strand of the transcripts was approximately 1.7∶1 for all libraries. This suggests that in spite of the high number of antisense mapping events detected, the transcriptional regulation in the N-PRRSV-induced immune response acts most strongly on the sense strand. To analyze the depth of transcriptome sampling in the DGE libraries, we studied the rate of increase of the number of genes (sense+antisense strand) identified as the size of the corresponding library increases. When the library size reached one million, we could identify 45% and 30% all genes and genes identified by unambigous tags, respectively (Figure S3). At this time, library capacity approached saturation.

### Identification of differentially expressed (DE) genes and signaling pathway analysis

To gain the global transcriptional changes in N-PRRSV infected porcine lungs, we applied the method described previously [27] to identify DE genes from the normalized DGE data by pairwise comparisons between all differential time points (N96/C, N168/C, N168/N96) during infection. Results showed that 5430 genes had p values <0.005, false discovery rate (FDR) <0.01 and estimated absolute log2-fold change >0.5 in at least one of the pairwise comparisons, which were declared to be differentially expressed during infection course (Table S3).

To characterize the functional consequences of gene expression changes associated with infection with N-PRRSV, we performed pathway analysis of DE genes based on the KEGG database by two-side Fisher's exact test. We chose only significant pathway categories that had a P-value of <0.05 and an FDR of <0.05. As shown in Figure S4, the significant signaling pathways include cell adhesion molecules (CAMs), T cell receptor signaling pathway, antigen processing and presentation, Toll-like receptor signaling pathway, biosynthesis of unsaturated fatty acids, pantothenate and CoA biosynthesis, etc (Table S4).

### Validation of DGE data by qPCR

**Figure 2:**
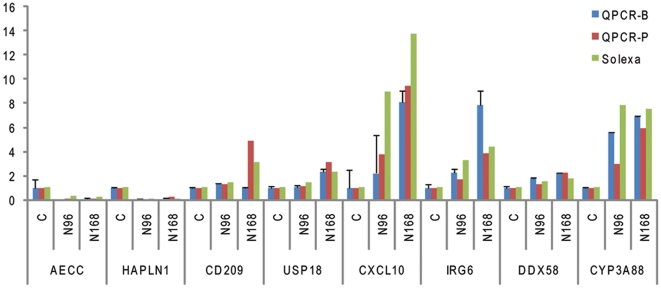
qPCR validation of DGE data. Relative quantitation was carried out to measure changes in target gene expression in lung samples relative to an endogenous reference sample. Results are expressed as the target/reference ratio of each sample normalized by the target/reference ratio of the calibrator. HPRT1 was used as a reference gene. The vertical axis indicates the fold change of transcript abundance in N-PRRSV infected pigs compared to the UNC. For the C sample, the fold change of transcript abundance relative to the C sample equals one, by definition. qPCR-B: the RNA samples from independent RNA extractions from biological replicates; qPCR-P:the RNA samples from pooling samples that were used for deep sequencing. Error bars represent SE.

To validate DE genes identified by Solexa sequencing, we selected 8 genes for qPCR confirmation. The set included two down-regulated genes (epithelial chloride channel protein (AECC) and hyaluronan and proteoglycan link protein 1 (HAPLN1)) and six up-regulated genes (inflammatory response protein 6 (IRG6), DEAD (Asp-Glu-Ala-Asp) box polypeptide 58 (DDX58), USP18, CXCL10, cytochrome P450 (CYP3A88), and CD209). Data were presented as fold changes in gene expression normalized to the HPRT1 gene and relative to the C sample. Pearson's correlation coefficient (r) showed that both the DGE and qPCR data (pooling samples) were highly correlated, for the genes modulated by N-PRRSV had a high consistency and r values ranging from 0.781 (CYP3A88) to 0.997 (AECC) between the two methods (Figure 2). qPCR analysis (both pooling samples and independent RNA extractions from biological replicates) confirmed the direction of change detected by DGE analysis. This correlation indicated the reliability of DGE results.

### Identification of DE genes in known pig Quantitative trait loci (QTL) regions associated with health traits

QTL play a central role in linking genomic information with phenotypes. The ultimate goal of QTL studies is to identificate the actual gene(s) that are responsible for the phenotypic variation observed in a particular trait [31]. In the present paper, we mapped the DE genes to pig QTL regions of health traits in pig QTLdatabase (PigQTLdb). Our search found that 240 DE genes were distributed in 18 different known QTL regions related to pig health traits (Figure S5; Table S5). Among the 240 DE genes, 122 and 114 were located in QTL regions of the CD4-Positive Leukocytes and CD2-Positive Leukocytes, respectively; 53 were distributed in the QTL region of the Band-Formed Neutrophils and CD8-Positive Leukocytes. Immune responses against pathogens depend in part on the generation of fully differentiated ‘killer’ (or effector) and memory CD8^+^ T cell. Effective priming and maintenance of CD8^+^ T cell responses to viral infection require ‘help’ from CD4^+^ T cells, the latter play also a critical role in programming CD8^+^ T cell memory development [32]. Moreover, recent study showed that CD4^+^ T cells guide effector cytotoxic T lymphocytes (CTLs) to virally infected tissues where they can destroy infected cells [32], [33].

### STC and STC-GO analysis

In order to profile gene expression time series and search for the most probable set of clusters generating the observed time series, we used STC algorithm of gene expression dynamics, which explicitly took into account the dynamic nature of temporal gene expression profiles during clustering and identified the number of distinct clusters. By STC, DE genes exhibited 8 types of temporal expression pattern (Figure S6; Table S3) with 4 (profile 1,6,0,7) significant cluster profiles which have significantly more genes assigned under the true ordering of time points compared to the average number assigned to the model profile in the permutation runs (Figure S6). Then Gene Ontology (GO) based on biological process (BP) enrichment analyses for sets of DE genes having significant cluster profiles was performed by two-side Fisher's exact test (Table S6 and Table S7; Figures S7, S8, S9 S10). We chose only significant GO categories that had a P-value of <0.05.

The most prominently overrepresented GO terms of significant cluster profile 1 (0,−1,−1) and profile 0 (0,−1,−2), which are down-regulated genes, involved in regulation of lipid, cholesterol biosynthetic and metabolic process; regulation of skeletal muscle development, muscle cell differentiation; digestion; negative regulation of neuron apoptosis and neurological system process (Table S6; Figures S7 and S8). The most prominently overrepresented GO terms of significant cluster profile 6 (0,1,1) and profile 7 (0,1,2), which are up-regulated genes, included negative regulation of fibroblast proliferation; natural killer cell, macrophage, lymphocyte, mononuclear cell, leukocyte and T cell proliferation, differentiation and activation; complement activation, immune response, inflammatory response, defense response, and apoptosis; response to stimulus(stress); lipid and fatty acid metabolic process and oxidation; positive regulation of ubiquitin-protein ligase activity and protein proteolysis, protein targeting to mitochondrion(Table S7; Figure S9 and S10). These results are consistent with these genes and their associated processes playing important roles in N-PRRSV replication and pathogenesis.

### N-PRRSV replication and spread

**Figure 3:**
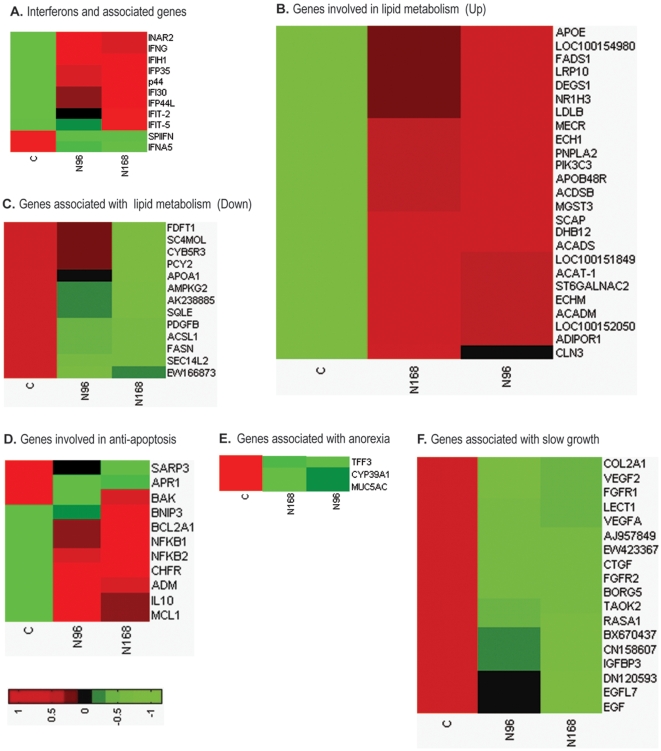
Differential expression genes related to the regulation of replication and spread of N-PRRSV, anorexia, slow growth. (A) Interferons and associated genes; (B) Upregulated genes involved in lipid metabolism; (C) Downregulated genes associated with sterol, cholesterol, and lipid biosynthetic and metabolic process; (D) Genes involved in the induction of anti-apoptotic state; (E) Genes associated with anorexia and (F) subsequent slow growth. Genes shown in red were upregulated and those shown in green were downregulated in infected relative to UNC pigs. See supplementary Table S3 for full gene names.

Viral infection of host leads to the initiation of antiviral innate immune responses, which results in the induction of expression of the type I interferons [34]. Meanwhile, many viruses have also developed strategies to evade and subvert the immune response. As shown in Figure 3A, transcripts of the IFN γ was significantly induced in N-PRRSV-infected pigs at days 4 through 7 pi, but short type I interferon (SPI IFN) gene expression was suppressed, and interferon alpha 5 (IFNA5) gene expression was markedly down-regulated.

Lipid rafts, lipid microdomains of the cell membrane enriched in sphingolipids, cholesterol and associated proteins, play critical roles in the life cycle of many viruses [35]. Some viruses enhance their replication by modulating host cell lipid metabolism [36]. DGE analysis of pigs infected with N-PRRSV showed significant increase of transcript abundance in many genes involved in lipid metabolism, including those for apolipoprotein B48 receptor (APOB48R), apolipoprotein-E (APOE), low density lipoprotein B (LDLB), phosphatidylinositol 3-kinase catalytic subunit type 3 (PIK3C3) (Figure 3B). Perhaps N-PRRSV alters hosts' lipid metabolism to create a lipid-rich intracellular environment to facilitate its own multiplication. Moreover, we also observed that N-PRRSV induced upregulation expression of anti-apoptotic genes in N-PRRSV infected lungs, including myeloid cell leukemia sequence 1 (BCL2-related) (MCL1), nuclear factor kappa-B 1 (NFKB1), NFKB2, adrenomedullin (ADM), and interleukin 10 (IL10), and downregulation expression of pro-apoptotic genes, including Bak protein (BAK), (apoptosis-related protein 1) (APR1) (Figure 3D) to inhibit apoptosis, which might prolong cell life and increase the yield of progeny virions.

N-PRRSV infection caused anorexia and subsequent slow growth. Accordingly, we observed that transcript abundance of genes involved in digestion, such as gastric mucin (MUC5AC) and cytochrome P450 (CYP39A1), was significantly decreased (Figure 3E). Simultaneously, transcript abundance of the genes associated with cell, muscle and cartilage development was markedly decreased (Figure 3F). These genes include insulin-like growth factor binding protein 3 (IGFBP3), collagen, type II, alpha 1 isoform 2 (COL2A1), connective tissue growth factor (CTGF), epidermal growth factor (EGF).

### Fever and heat shock

**Figure 4:**
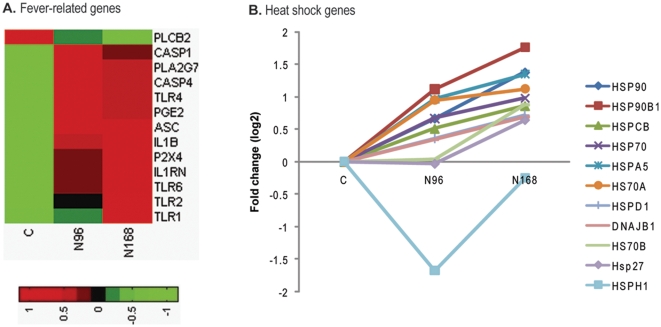
Differential expression in functional processes related to fever. (A) Fever-related genes; (B) Heat shock genes. The red and green color represent significantly induced or repressed gene expression, respectively. See supplementary Table S3 for full gene names.

Fever is frequently the host's initial response to infection [37]. After viral infection, pathogen-associated molecular patterns (PAMPs) in viral proteins and nucleic acids were recognized by host pathogen-recognition receptors (PRRs), such as Toll-like receptors (TLRs), which trigger gene expression and synthesis of the IL-1β precursor. Active caspase-1 (CASP1) cleaves the IL-1β precursor into mature, bioactive IL-1β, which is an inflammatory cytokine most responsible for fever [37], [38], [39]. As shown in Figure 4A, transcript abundance of TLR1, 2, 4, 6, IL-1β and CASP1 was significantly increased in N-PRRSV infected porcine lungs. Moreover, transcript abundance of genes involved in the activation of CASP1 and IL-1β secretion including apoptosis-associated speck-like protein containing a CARD (ASC), prostaglandin E synthase 2 (PGE2) and phospholipase A2, group VII (PLA2G7) was significantly increased (Figure 4A). The expression of heat shock proteins (HSPs), known as stress proteins, can be markedly upregulated by all cells under conditions of stress, such as increased temperature (fever) and viral infection [40]. Transcript abundance for most of these heat shock genes, including 90-kDa HSP (HSP90), HSP70, and heat shock protein beta-1 (Hsp27) was significantly elevated in N-PRRSV infected lungs relative to UNC lungs (Figure 4B).

### Inflammatory response and interstitial pneumonia

**Figure 5:**
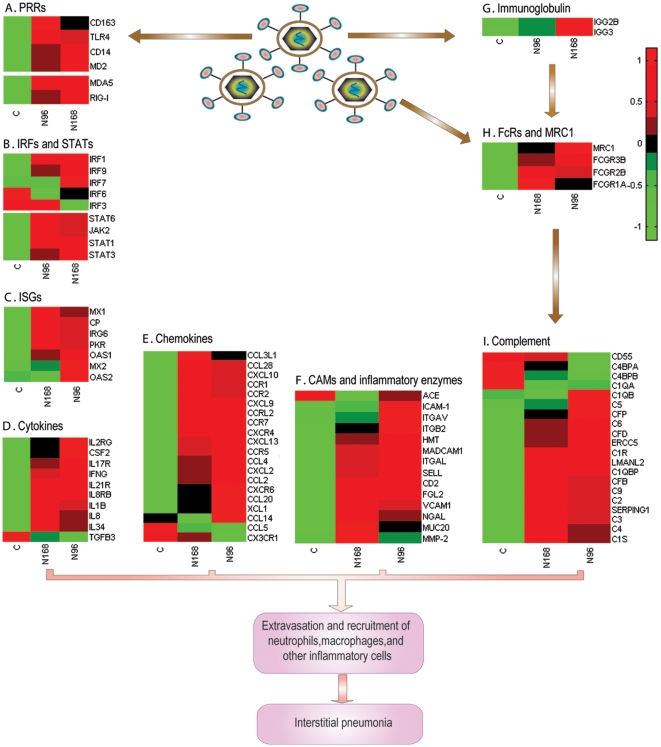
Expression of genes involved in inflammatory response in N-PRRSV-infected porcine lungs. (A) Cell surface and cytoplasmic pattern-recognition receptors (PRRs); (B) IFN-regulatory factors (IRFs) and signal transducer and activator of transcription (STATs); (C) Interferon-stimulated genes (ISGs); (D) Cytokines; (E) Chemokines; (F) Adhesion molecules and inflammatory enzymes; (G) Immunoglobulin; (H) Fc receptors and mannose receptor C1 (MRC1); (I) Complement. See supplementary Table S3 for full gene names.

Viral infection results in an inflammatory response, which is an essential component of the antiviral innate immune response [41]. After recognizing the PAMPs, either surface or intracellular PRRs trigger intracellular signaling cascades that results in the activation of transcription factors, including nuclear factor-κB (NF-κB), interferon-regulatory factors (IRFs), and signal transducer and activator of transcription (STATs). As shown in Figure 4A, transcripts of the Toll-like PRRs TLR1, TLR2, TLR4, TLR6, were significantly increased in N-PRRSV-infected pigs at days 4 through 7 pi, but no change in TLR3 which specializes in the recognition of viral dsRNA was detected. Cytoplasmic PRRs (Figure 5A), retinoic-acid-inducible protein I (RIG-I, DDX58) and melanoma differentiation-associated gene 5 (MDA5), the two most relevant for defense against viruses, were expressed at a high level after N-PRRSV infection. Cell surface PRRs such as CD14, MD-2 protein (MD2) and CD163 (which is probably involved in PRRSV entry during uncoating [42]) were likewise up-regulated expression after N-PRRSV infection (Figure 5A). After binding to N-PRRSV viral PAMPs, PRRs initiate intracellular signaling cascades that activate transcription factors, including IRF1, IRF7, IRF9, but not IRF3 and STAT1, STAT3, STAT6 (Figure 5B). Activated transcription factors and STATs in turn induce the transcription of specific sets of interferon-stimulated genes (ISGs) [34], [43], and expression of multiple inflammatory genes [44], which induce a pro-inflammatory response and attract cells, such as neutrophils and macrophages, to sites of infection. Accordingly, we observed significant increase of transcript abundance in many genes involved in ISGs (Figure 5C), pro-inflammatory cytokines (such as IL1β, IL8) (Figure 5D), chemokines (CCL2, CXCL9, CXCL10) (Figure 5E), adhesion molecules (VCAM, ICAM1, SELL), and other inflammatory molecules (such as MMP-2,) (Figure 5F). Moreover, immunoglobulin (such as IGG2B, IGG3) (Figure 5G), three categories of Fc receptors and mannose receptor C1 (MRC1) (Figure 5H), and complement proteins (Figure 5I) were also significantly induced in the N-PRRSV-infected lungs. However, several complement inhibitors that possess inhibitory and/or decay-accelerating acitivity, such as decay-accelerating factor CD55, complement component 4 binding protein, alpha (C4BPA), C4BPB, were significantly repressed in the N-PRRSV-infected lungs (Figure 5I).

### Cell death and tissues damage

**Figure 6:**
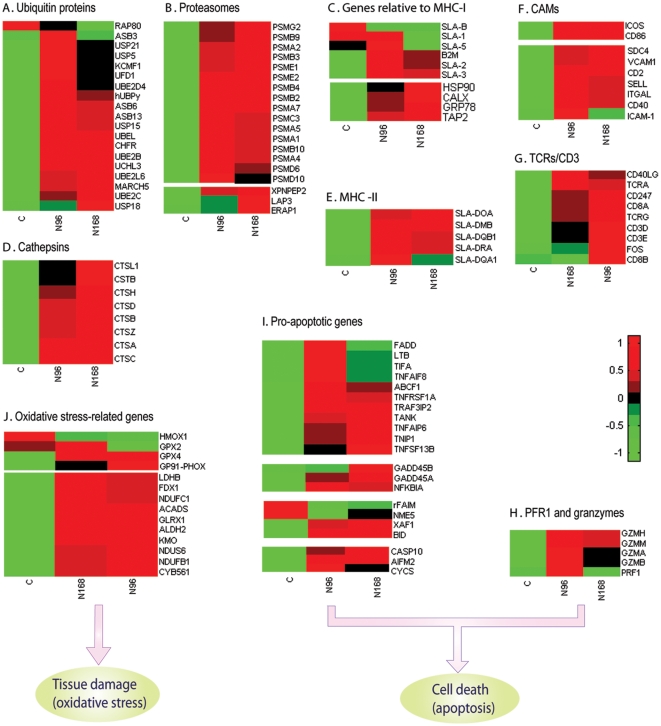
Expression of selected cell death and tissues damage -related genes in N-PRRSV-infected porcine lungs. (A) Ubiquitin-proteins and ubiquitin enzymes; (B) Proteasomes; (C) Genes relative to assembling and transport of MHC-I-peptide complex; (D) Cathepsins; (E) MHC class II antigens; (F) Costimulatory molecules and cell adhesion molecules (CAMs); (G) TCRs/CD3 complex and co-receptor molecules;(H) Perforin (PFR) and granzymes; (I) Pro-apoptotic genes; (J) Oxidative stress-related genes. See supplementary Table S3 for full gene names.

Cytotoxic T lymphocytes (CTLs) detect cells infected with a virus and destroy them through perforin-mediated apoptosis [45]. CD8^+^ T cells activation require T cell receptors (TCRs) to recognize cognate antigenic peptides for presentation on MHC class I molecules displayed on the surface of antigen presenting cells (APCs) [32]. Accordingly, we observed that transcript abundance of ubiquitin specific peptidase (USP) and ubiquitin enzyme (Figure 6A), 16 proteasomes, and aminopeptidases (Figure 6B) was significantly increased in N-PRRSV-infected lungs. The transcript abundance of B2M, MHC class I antigen 2 (SLA-2), SLA-3, TAP2, and chaperones (such as GRP78) was markedly increased after infection with N-PRRSV while the transcript abundance of SLA-B was significantly decreased (Figure 6C). In addition to recognization of cognate peptides presented by MHC class I molecules, CD8^+^ T cells activation needs also to receive ‘costimulatory’ signals and help by helper CD4 T^+^ cells [33]. As shown in Figure 6D and 6E, 8 cathepsins and 5 MHC class II antigens were significantly induced in N-PRRSV-infected lungs. The transcript abundance of costimulatory molecules (such as CD86, ICOS), CAMs, and TCRs/CD3 complex as well as co-receptor molecules (such as CD8A, CD8B) was remarkably increased after infection with N-PRRSV (Figure 6F and 6G). Activated CTLs release perforin (PFR) and granzymes, which two effectors act collaboratively to induce apoptosis of target cells. As shown in Figure 6H, PRF1 and granzyme B (GZMB) transcript abundance was significantly elevated in N-PRRSV infected lungs relative to UNC lungs. In addition to cytotoxins released from CTLs, the transcript abundance of other pro-apoptotic members (Figure 6I), such as NFKBIA, growth arrest and DNA-damage-inducible protein alpha (GADD45A), BH3 interacting domain death agonist (BID), XIAP-associated factor 1 (XAF1), cytochrome c (CYCS), CASP10, was also significantly increased after infection with N-PRRSV, which can induce apoptosis of virus-infected cells. In addition, we also identified the upregulated expression of cytochrome b245 heavy chain (GP91-PHOX), a critical component of the membrane-bound oxidase of phagocytes (macrophages and neutrophils), and the downregulated expression of heme oxygenase 1 (HMOX1) during N-PRRSV infections, which might result in the oxidative stress response and subsequent oxidative damage of tissues (Figure 6J).

### Relationship between pulmonary gene expression profiles and N-PRRSV infection pathology

**Figure 7:**
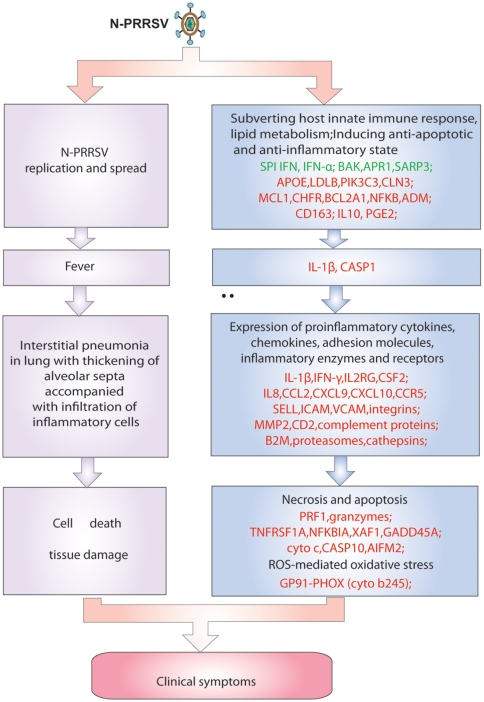
Model of the relationship between pulmonary gene expression profiles and infection pathology. Genes shown in red were upregulated and those shown in green were downregulated in infected relative to UNC pigs.

From the data presented in the paper, a model for the relationship between pulmonary gene expression profiles and infection pathology can be surmised in Figure 7, N-PRRSV virus replicates and spreads by subverting host innate immune response and hijacking host lipid metabolism as well as inducing an anti-apoptotic and anti-inflammatory state, as indicated by suppression expression of SPI IFN, IFN-α, down-regulation expression of pro-apoptotic genes for BAK, APR-1, SARP3, high levels expression of genes involved in lipid metabolism, such as APOE, LDLB, PIK3C3, anti-apoptotic genes for MCL1, BCL2A1, CHFR, ADM, NFKB, IL10, and anti-inflammatory molecule PGE2 as well as CD163. Infections of N-PRRSV viruses resulted in fever and inflammatory response, as indicated by high expression of proinflammatory cytokines and chemokines, adhesion molecules, inflammatory enzymes and receptors, such as IL-1β, IL8, SELL, ICAM, CCL2, CXCL9, CXCL10, B2M, proteasomes, cathepsins. This was compounded by cell death and elevated expression of NFKBIA, XAF1, GADD45A, perforin, granzymes, and cytochrome c, coupled with increased ROS-mediated oxidative stress, as indicated by by up-regulation expression of cytochrome b245. Taken together, the N-PRRSV infections may have resulted in an excessively immune and inflammatory response that contributed to tissue damage.

## Discussion

Infection of pigs with N-PRRSV caused fever, dyspnoea, reddening of skin, oedema of the eyelids, conjunctivitis, depression, anorexia, mild diarrhoea. Histopathology examination showed interstitial pneumonia in lungs with thickening of alveolar septa accompanied with infiltration of immune cells [46] (Figure 1). Although great efforts have been made by many researchers, the molecular basis of N-PRRSV infection is largely unknown. Here we report on the first genome-wide host transcriptional response to N-PRRSV infection using Solexa/Illumina's digital gene expression (DGE) system, a tag-based novel high-throughput transcriptome deep sequencing method. Given the nature of the methodology of Illumina's DGE system, we have pooled biological replicates from three pigs for each group to make representative samples for deep sequencing analysis. We could reach a sequencing depth of 6.3–7.9 million tags per library (Table 1) and found over 5000 genes to be differentially expressed during N-PRRSV infection processes (Table S3). Although others studies have also pooled biological replicates for library construction and deep sequencing [47], [48], resulting in the lack of biological replicate, one may blur the impact of variations in pooling samples. Because of the variations of pigs in response to PRRSV infection, it is possible that one pig could significantly affect results without independent libraries. But we performed the qPCR validation both on the same pooled material that was used for deep sequencing and on independent RNA extractions from each pig, which all confirmed the direction of change detected by DGE analysis (Figure 2).

Our DGE analysis showed massive changes in the transcript abundace of known immune response genes and of genes that have been implicated in PRRSV infection [18], [19], [20]. We also identified many interesting genes that had not been linked to PRRSV infection in previous studies. For example, transcript abundace of lipid metabolism-related genes including APOB48R, APOE, PIK3C, was significantly increased during N-PRRSV infection processes. Alterations in lipid metabolism, perhaps including those with significant upregulation in this study, have been observed in response to infection by a range of viruses including SARS-CoV, HCV, influenza A virus, or dengue virus [35], [36], [49], [50]. Although *In vitro* studies have investigated how PRRSV modifying genes expression in PAMs [18], [19], [20], many of the outstanding issues will be answered only in the context of PRRSV-infected animals [51]. Hence, we characterized the genome-wide transcriptome response to PRRSV infection in porcine lung by deeping sequencing. But studies of transcript abundance in lung tissues have also their intrinsic limitations. For example, the transcriptome of lung tissues is actually a merging transcriptional responses of a wide range of cell types, some of which are infected, some of which are responding directly to the infectious process and others of which are bystanders. Moreover, increased cellularity of tissues may be confused as biologically important increased transcript abundance. Despite such limitations, our DGE study offers a broad, system-wide window into molecular processes that regulate gene expression and also provides new leads for functional studies of candidate genes involved in host-virus interaction, as illustrated in this paper.

The induction of expression of type I interferons (IFNs; including IFN-α and IFN-β) is a well-known innate antiviral immune reaction in the virus-infected cells [52], [53]. However, N-PRRSV infection suppressed SPI IFN gene expression and decreased the transcript abundance of IFN-α (Figure 3A). Previous studies [19], [54], [55], both in vitro and in vivo, have also showed that PRRSV elicited only a minimal IFN-α production or even suppressed it's expression. The suppression of SPI IFN, in particular of IFN-α, is probably a crucial step in the pathogenesis, because IFN-α has been shown to inhibit PRRSV replication [11]. Other viruses infection, such as the 1918 influenza virus [56], hepatitis C virus (HCV) [57], Ebola virus [58], also suppressed type I IFN gene expression which led to extensive viral replication and increased pathogenesis. IRF3 plays an important role for type I IFN gene expression. The transcript abundance of IRF3 was decreased intensively in N-PRRSV-infected pigs by 168 h pi (Figure 4B). One study [59] indicated that PRRSV NSP1β inhibited IRF3, and then down-regulated IFN-β gene expression. It is worth mentioning that the NSP1 of the influenza A can also suppress innate immunity by inhibiting IRF3 activation, and subsequently disrupting the induction of α/β–interferon [60]. Research has indicated that the expression of CD163, a PRRSV receptor [61], on macrophages in different microenvironments, *in vivo*, may determine the replication efficiency and subsequent pathogenecity of PRRSV [62]. Transcript abundance of CD163 was significantly increased after N-PRRSV infection (Figure 5A). The internalization of PRRSV via CD163 in the target cells may induce the expression of IL10, and in turn induce the expression of CD163 on neighboring undifferentiated monocytes and increased overall PRRSV susceptibility [62].

Moreover, infected pigs develop a strong and rapid humoral response but these initial antibodies do not confer protection and can even be harmful by mediating an ADE [12]. These antibodies enhance PRRSV viral replication by coating the virus and enhancing the internalization of viral particles into macrophages [12]. As shown in Figure 5G and 5H, IgG and Fcγ receptors were significantly induced during N-PRRSV infection processes. Interestingly, the presence of antibodies during feline enteric coronaviruses (FECVs) infection does not also provide sterilizing immunity, instead, these antibodies opsonize virus particles and facilitate their entry to monocytes and/or macrophages through Fcγ receptors, resulting in disease enhancement [51].

The activation of pro-inflammatory transcription factor NF-κB induces robust activation of the CASP1 inflammasome and subsequent release of IL-1β that cause fever and inflammation [63], [64], [65], [66]. Accordingly, we identified upregulation expression of CASP1, NF-κB, and IL-1β genes during N-PRRSV infection processes (Figure 4A). NF-κB activation also enhanced the expression of matrix metalloproteinases (MMP2) and MMP9, two cytotoxic substances in PRRSV-infected cells [11], [67]. Similarly, transcript abundance of MMP2 and NGAL (25 kDa alpha-2-microglobulin-related subunit of MMP-9) was significantly increased in the lungs of N-PRRSV-infected pigs (Figure 5F). Upregulation expression of MMPs would likely facilitate infiltration of inflammatory cells and increase inflammation.

Upregulation expression of IL8 (also known as CXCL8), which is an attractant for neutrophils and other polymorphonuclear leukocytes produced after acute infection, in PRRSV-infected PAMs [68], [69] and lungs of N-PRRSV-infected pigs (Figure 5D), was observed. Other chemokines such as CCL2 (also known as MCP1 ), CXCL9, CXCL10 (also known as IP10), which were significantly increased (Figure 5D), may be also crucial for lymphocyte and macrophage infiltration into the sites of N-PRRSV infection. CCL2, IL8 and IP10 expression were upregulated during SARS-CoV [70], [71], and murine coronavirus [72] infections process, which may recruit monocytes and/or macrophages to sites of infection and be a major cause of lung pathology. Although the present study indicates that upregulation expression of pro-inflammatory molecules contributes to the pathogenesis of N-PRRSV, increased transcript abundance of anti-inflammatory molecules, such as IL10, PGE2, was also detected in the study. Upregulation of IL10 gene expression was found previously in PRRSV-infected porcine leukocytes, PAMs, DCs, and *in vivo* in PRRSV infected pigs [16], [17], [19], [73]. Perhaps an increase in pro-inflammatory molecules followed by increased anti-inflammatory molecules is the normal progression of events in inflammation [74]. The upregulation expression of IL10 might skew the immune response away from a protective Th1-cell response towards a non-protective Th2-cell response, therefore impairing clearance of virus, which benefits viral infections [51]. Upregulation expression of anti-inflammatory molecules and pro-inflammatory molecules occurring concurrently was also observed after SARS-CoV and FIPV infection [75], [76]. Antibodies might also contribute to immunopathogenesis through increasing the uptake of virus by macrophages, resulting in activation of these macrophages and secretion of pro-inflammatory cytokines and chemokines. Antigen-antibody complexes might increase transcript abundance of complement (Figure 5I), which leads to generation of the classical inflammatory response through the production of potent proinflammatory molecules [77]. Furthermore, complement activation might also contribute to the development of pulmonary edema and oedema of the eyelids. Further understanding the roles complement plays in the host-pathogen interactions may help to develop more effective pharmacological agents against infection. Moreover, damage to the lungs of N-PRRSV-infected pigs seems to occur directly by viral destruction of alveolar and bronchial epithelial cells and macrophages (Figure 1C), as well as indirectly through production of immune mediators.

Activated CTLs and NK cells release perforin (PFR) and granzymes, which two effectors act collaboratively to induce apoptosis of target cells. Transcript abundance of PFR1 and granzymes increased in the lungs of N-PRRSV infected pigs (Figure 6H). Pro-apoptotic molecules XAF1, BID, cyto c, CASP10, AIFM2, were significantly up-regulated after infection with N-PRRSV, which may induce apoptosis of virus-infected cells (Figure 6I). Simultaneously, we also observed upregulation expression of anti-apoptotic genes in N-PRRSV infected lungs, including BCL2A1, MCL1, CHFR, NFKB, ADM, IL10 etc (Figure 3D). Upregulation expression of anti-apoptotic genes and pro-apoptotic genes occurring concurrently after N-PRRSV infection seems in contradiction of each other. However, this may reflect a balance between apoptotic and anti-apoptotic mechanisms. Perhaps PRRSV actively induces an anti-apoptotic state to complete its virus replication cycle through delaying cell death while induces apoptosis of virus-infected cells after completion of virus replication to increase rate of spread of virus [19], [78], [79]. Anti-apoptotic and pro-apoptotic activaties were also observed in PRRSV-infected Marc-145 cells, in which PRRSV stimulated anti-apoptotic pathways early in infection while caused apoptosis of PRRSV-infected cells late in infection [80], [81]. Infection with N-PRRSV also increased transcript abundance of NFKBIA (Figure 6I), an inhibitor of the TNF receptor activated transcription factor NF-κB. Loss of NF-κB activity has been shown to increase the cytotoxic effects of TNF which resulted in increased cell death [82]. An increase of transcript abundance in pro-apoptotic genes might result in disruption of the mitochondria transmembrane potential, thereby inducing release of cyto c from mitochondrial membranes to induce apoptosis and secondary necrosis [83].

The production of ROS, especially superoxide radicals, and the subsequent oxidative damage of cells and tissues are recognized as key contributors to the viral pathogenesis [82], [84]. ROS-mediated oxidative stress might also be involved in inducing apoptosis by PRRSV [81]. Accordingly, we identified the remarkable upregulation of cytochrome b245 heavy chain (GP91-PHOX) (Figure 6J), a critical component of the membrane-bound oxidase of phagocytes (macrophages and neutrophils), after infection with N-PRRSV, that generated superoxide radicals that killed both infected and normal cells at sites of infection, which would further exacerbate the immunopathological response.

Infection of macrophages, monocytes and DCs that are essential for immune function, is likely to be a key component in N-PRRSV-induced pathogenesis [19], [85], [86], [87]. Apoptosis of infected cells causes immune suppression by two mechanism: apoptosis either induces a decrease in the numbers of immune cells that compromises both the innate and adaptive immune response in which they are unable to eradicate the primary infection, or impairs immunity by inducing immunosuppressive effects in the surviving cells [88]. For example, uptake of apoptotic cells by normal macrophages and DCs stimulates immune tolerance by inducing the release of anti-inflammatory cytokines, such as IL10, and suppressing the release of pro-inflammatory cytokines [89]. Histopathological analysis of the lymphnodes of pigs infection with N-PRRSV revealed a profound depletion of immune cells compared with those of UNC (data not shown).

In summary, the data presented in this study suggest that N-PRRSV appears to utilize multiple strategies for its replication and spread in infected pigs, including subverting host innate immune response, inducing an anti-apoptotic and anti-inflammatory state as well as developing ADE. After infection of macrophages and possibly DCs, PRR-PAMP interactions triggered signaling cascades that increased the transcript abundance of multiple inflammatory molecules, including cytokines, chemokines, adhesion molecules and inflammatory enzymes that induce a pro-inflammatory response, activate and recruit immune cells, such as macrophages and neutrophils, to sites of infection for virus elimination and thereby produce the clinical symptoms of viral infection, such as fever, dyspnoea, interstitial pneumonia in lungs. Further, antibodies and complement activation might exacerbate inflammatory response. N-PRRSV might induce an immunosuppressive state, mediated by apoptosis of infected cells, which caused depletion of immune cells and induced an anti-inflammatory cytokine response in which they were unable to eradicate the primary infection.

## Supporting Information

Figure S1 Saturation of DGE libraries. Saturation analysis of capacity of libraries showed that new emerging distinct tags were gradually reduced with increasing of total sequence tags when the number of sequencing tags was big enough. (A) C; (B) N96; (C) N168.
(3.70 MB TIF)

Figure S2 The positions of tags in the gene. Ideally the tag is the 3 most one. But for alternative splicing or incomplete enzyme digestion, the tag may be the 2nd or 3rd from the 3 most one. (A) C; (B) N96; (C) N168.
(4.03 MB TIF)

Figure S3 Effect of library size on the number of gene identified. The rate of increase of all genes identified and genes identified by unambigous tags declined drastically as the size of the library increased. When the library size reached one million, we could identify 45% and 30% all genes and genes identified by unambigous tags, respectively. At this time, library capacity approached saturation. (A) C; (B) N96; (C) N168.
(1.05 MB TIF)

Figure S4 Signaling pathways of DE genes. Pathway analysis was mainly based on the KEGG database. A P-value of <0.05 and an FDR of <0.05 in the two-side Fisher's exact test were selected as the significant criteria. The vertical axis is the pathway category and the horizontal axis is the log10(p Value) of these significant pathways.
(0.66 MB TIF)

Figure S5 Genes that distributed in the known pig QTLs of Health Traits. The X axis represents the QTL symbol, and the Y axis indicates the number of genes associated with Health Traits. See Table S4 for full QTL names.
(0.60 MB TIF)

Figure S6 STC (Series Test of Cluster) analysis of DE genes. Dynamic gene expression profiles in all 5430 DE genes are shown for eight clusters (A). The eight profiles were ordered based on the p value significance of number of genes assigned versus expected. The upper left represents the serial number of the cluster, and the under left represents the p value. (B–E) four significant cluster profiles which have significantly more genes assigned under the true ordering of time points compared to the average number assigned to the model profile in the permutation runs. Y axis indicates the relative gene expression change presented in log2 ratio between UNC lung and N-PRRSV infected lungs at the indicated time points. (B) profile 1 (0,−1,−1), 595.3 genes were expected, but 1373.0 genes were assigned, p-value = 1.0E-191; (C) profile 6 (0,1,1), 810.7 genes were expected, but 1520.0 genes were assigned, p-value = 3.0E-134; (D) profile 0 (0,−1,−2), 646.0 genes were expected, but 404.0 genes were assigned, p-value = 2.8E-31; (E) profile 7 (0,1,2), 456.0 genes were expected, but 404.0 genes were assigned, p-value = 4.4E-3.
(3.73 MB TIF)

Figure S7 Biological process GO terms of profile 1. Functional classification of the DE genes was performed according to GO biological processes. A P-value of <0.05 in the two-side Fisher's exact test were selected as the significant criteria. These DE genes were sorted by the enrichment of GO categories. The vertical axis is the GO category and the horizontal axis is the enrichment of GO.
(1.53 MB TIF)

Figure S8 Biological process GO terms of profile 0. Functional classification of the DE genes was performed according to GO biological processes. A P-value of <0.05 in the two-side Fisher's exact test were selected as the significant criteria. These DE genes were sorted by the enrichment of GO categories. The vertical axis is the GO category and the horizontal axis is the enrichment of GO.
(2.78 MB TIF)

Figure S9 Biological process GO terms of profile 6. Functional classification of the DE genes was performed according to GO biological processes. A P-value of <0.05 in the two-side Fisher's exact test were selected as the significant criteria. These DE genes were sorted by the enrichment of GO categories. The vertical axis is the GO category and the horizontal axis is the enrichment of GO.
(1.44 MB TIF)

Figure S10 Biological process GO terms of profile 7. Functional classification of the DE genes was performed according to GO biological processes. A P-value of <0.05 in the two-side Fisher's exact test were selected as the significant criteria. These DE genes were sorted by the enrichment of GO categories. The vertical axis is the GO category and the horizontal axis is the enrichment of GO.
(1.56 MB TIF)

Table S1 QPCR results of N-PRRSV NSP2 gene expression in infected pigs.
(0.03 MB DOC)

Table S2 Summary of antisense transcripts.
(8.24 MB XLS)

Table S3 Summary of differentially expressed (DE) genes identified.
(1.83 MB XLS)

Table S4 Pathway analysis of DE genes.
(0.11 MB XLS)

Table S5 Health traits of QTL associated with DE genes.
(0.13 MB XLS)

Table S6 Series Test Cluster of Gene Ontology (STC-GO) analysis of profile 1 and 0.
(0.97 MB XLS)

Table S7 Series Test Cluster of Gene Ontology (STC-GO) analysis of profile 6 and 7.
(1.33 MB XLS)
