# Supplementary figures and images for: Understanding PRRSV Infection in Porcine Lung Based on Genome-Wide Transcriptome Response Identified by Deep Sequencing
Source: PLoS One. 2010 Jun 29;5(6):e11377. doi: 10.1371/journal.pone.0011377 (PMC2894071; doi:10.1371/journal.pone.0011377)

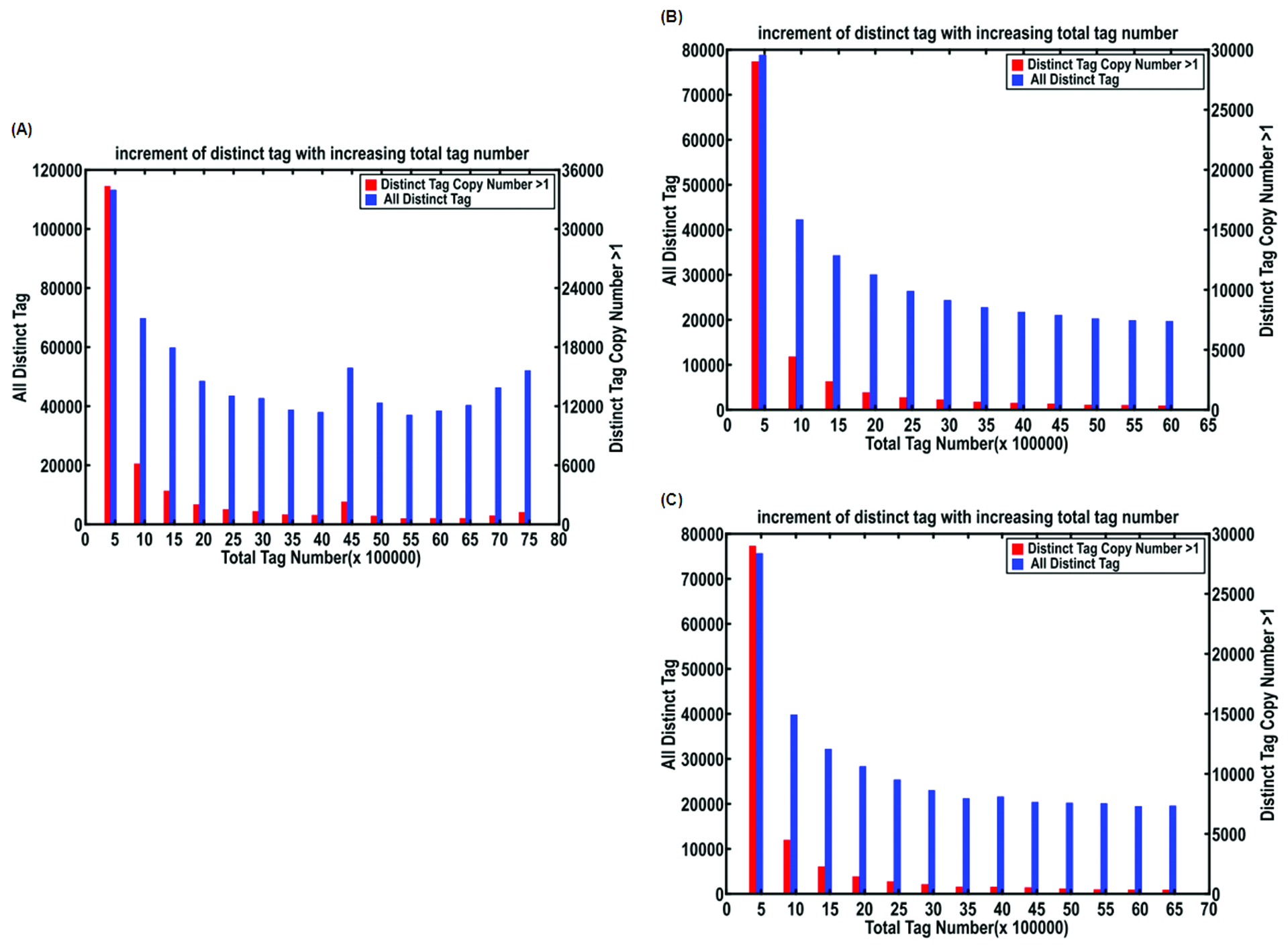

Supplement: Figure S1 — Saturation of DGE libraries. Saturation analysis of capacity of libraries showed that new emerging distinct tags were gradually reduced with increasing of total sequence tags when the number of sequencing tags was big enough. (A) C; (B) N96; (C) N168. (3.70 MB TIF) [file pone.0011377.s001.tif]

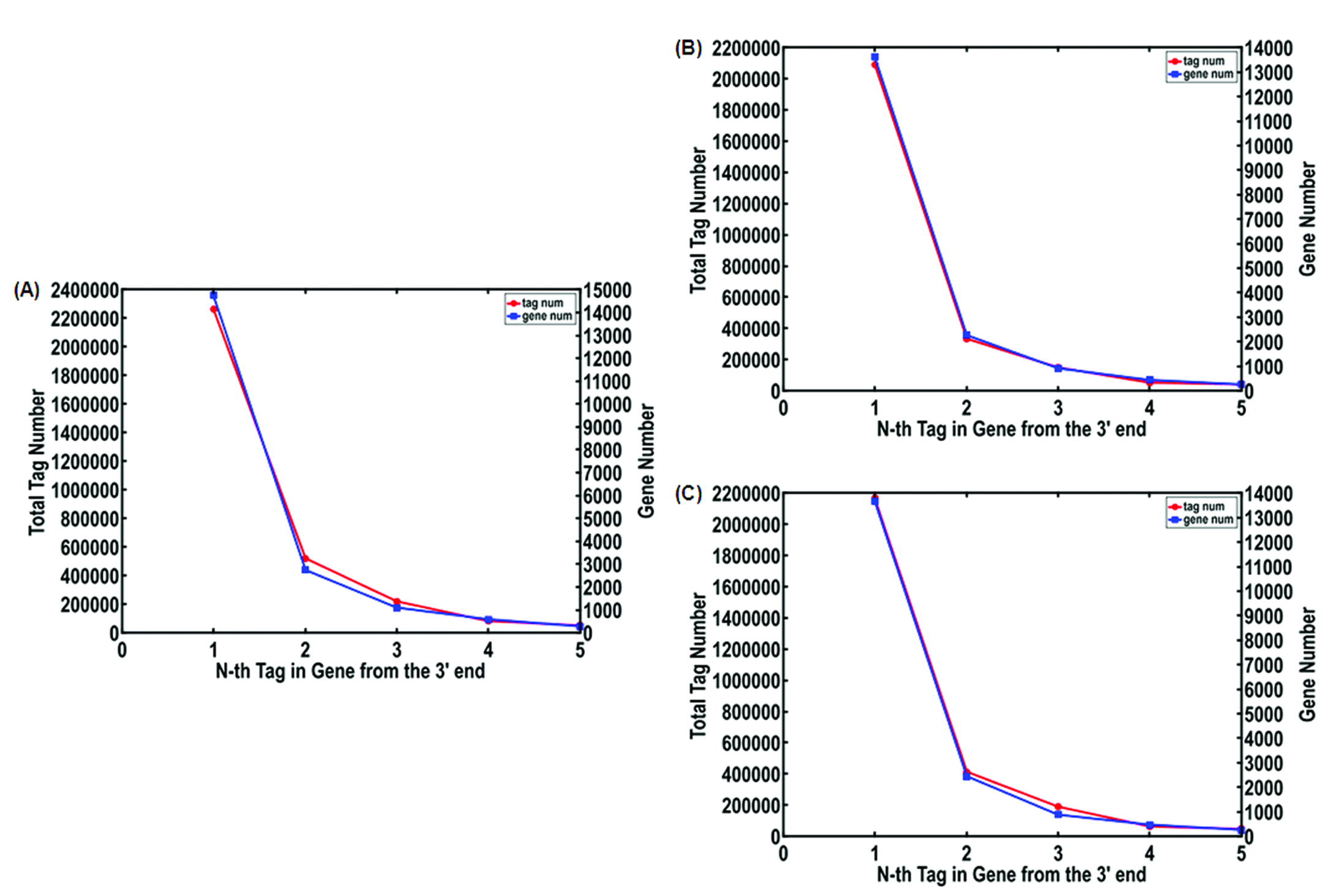

Supplement: Figure S2 — The positions of tags in the gene. Ideally the tag is the 3 most one. But for alternative splicing or incomplete enzyme digestion, the tag may be the 2nd or 3rd from the 3 most one. (A) C; (B) N96; (C) N168. (4.03 MB TIF) [file pone.0011377.s002.tif]

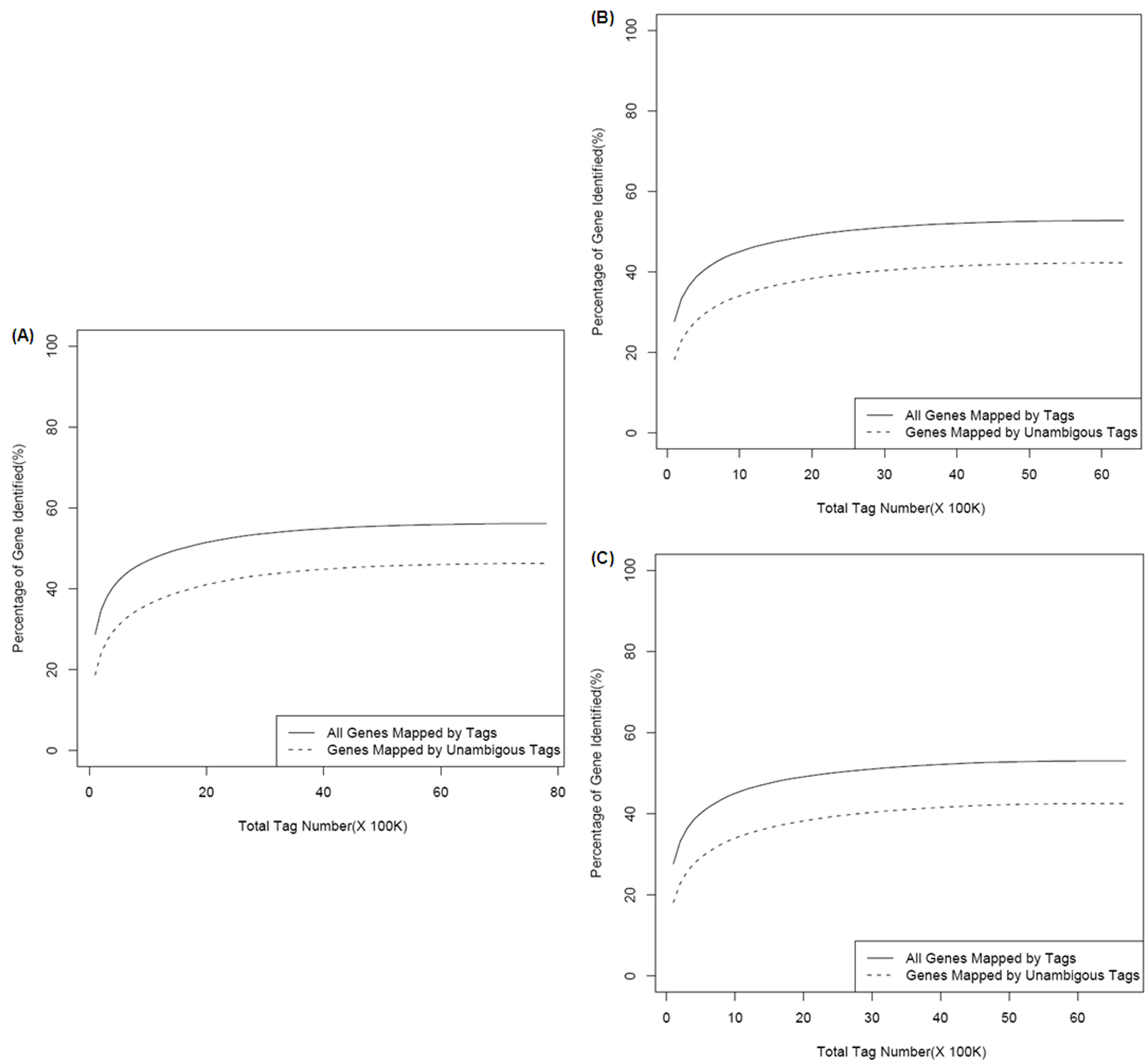

Supplement: Figure S3 — Effect of library size on the number of gene identified. The rate of increase of all genes identified and genes identified by unambigous tags declined drastically as the size of the library increased. When the library size reached one million, we could identify 45% and 30% all genes and genes identified by unambigous tags, respectively. At this time, library capacity approached saturation. (A) C; (B) N96; (C) N168. (1.05 MB TIF) [file pone.0011377.s003.tif]

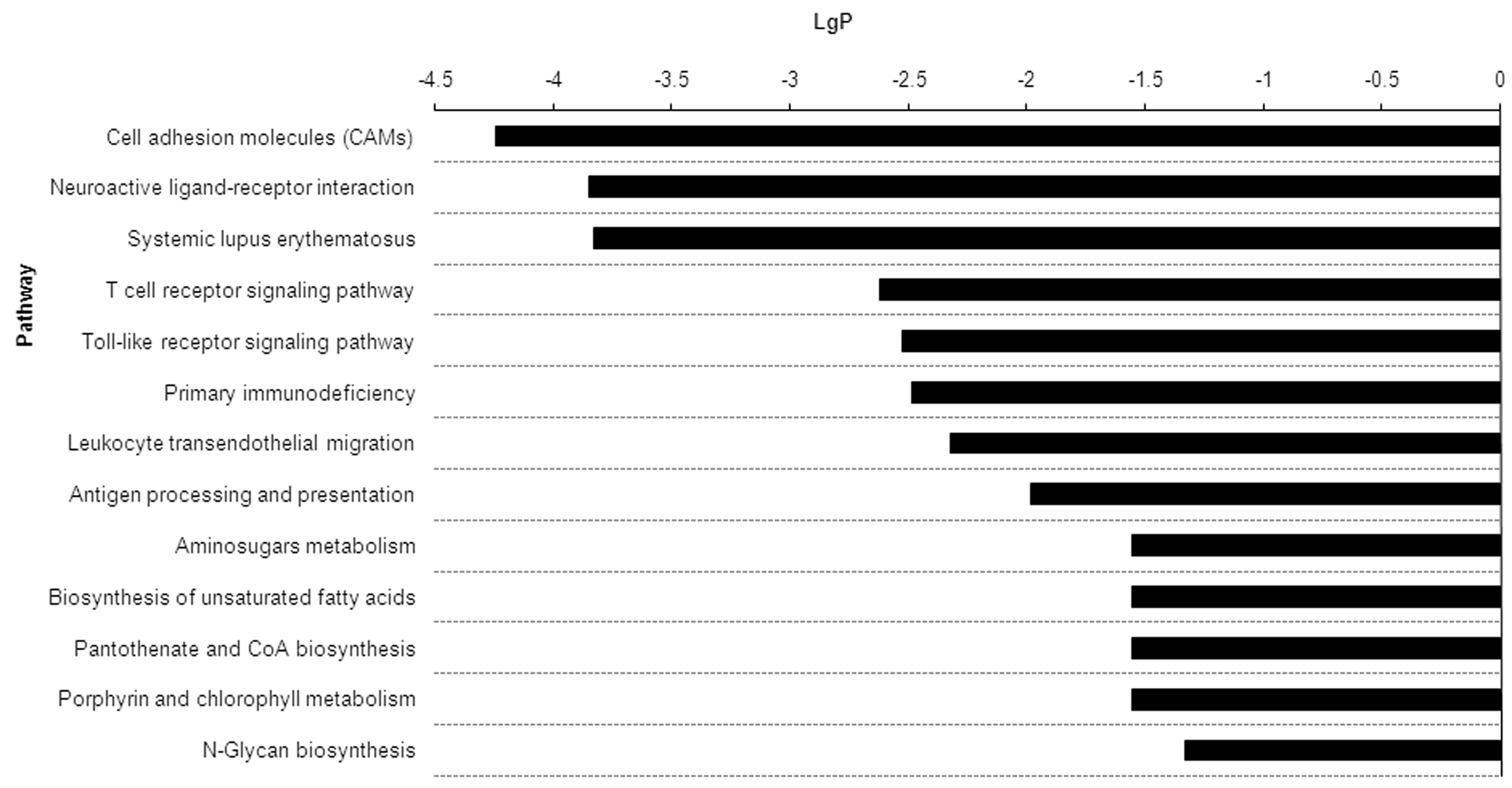

Supplement: Figure S4 — Signaling pathways of DE genes. Pathway analysis was mainly based on the KEGG database. A P-value of <0.05 and an FDR of <0.05 in the two-side Fisher's exact test were selected as the significant criteria. The vertical axis is the pathway category and the horizontal axis is the log10(p Value) of these significant pathways. (0.66 MB TIF) [file pone.0011377.s004.tif]

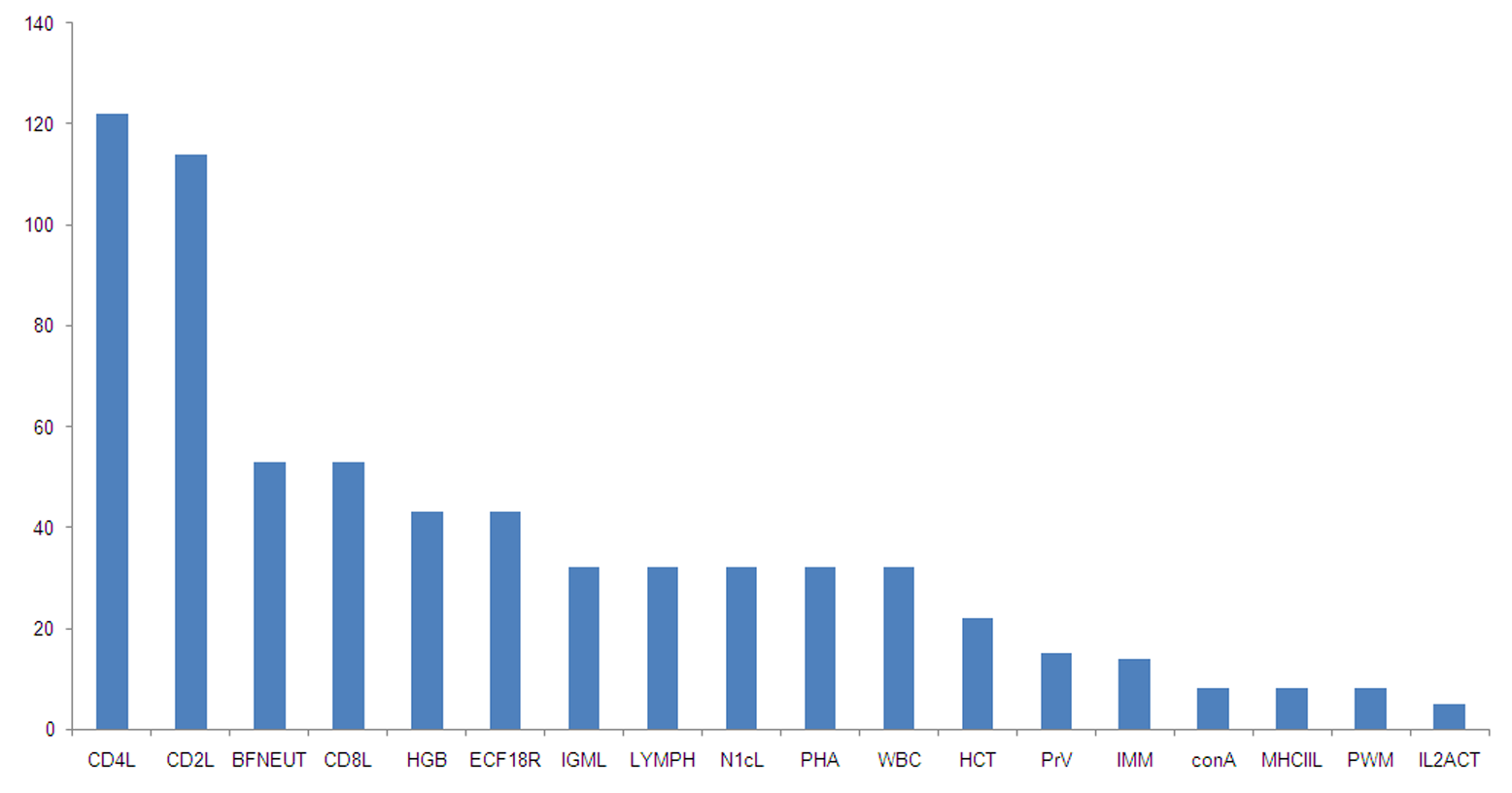

Supplement: Figure S5 — Genes that distributed in the known pig QTLs of Health Traits. The X axis represents the QTL symbol, and the Y axis indicates the number of genes associated with Health Traits. See Table S4 for full QTL names. (0.60 MB TIF) [file pone.0011377.s005.tif]

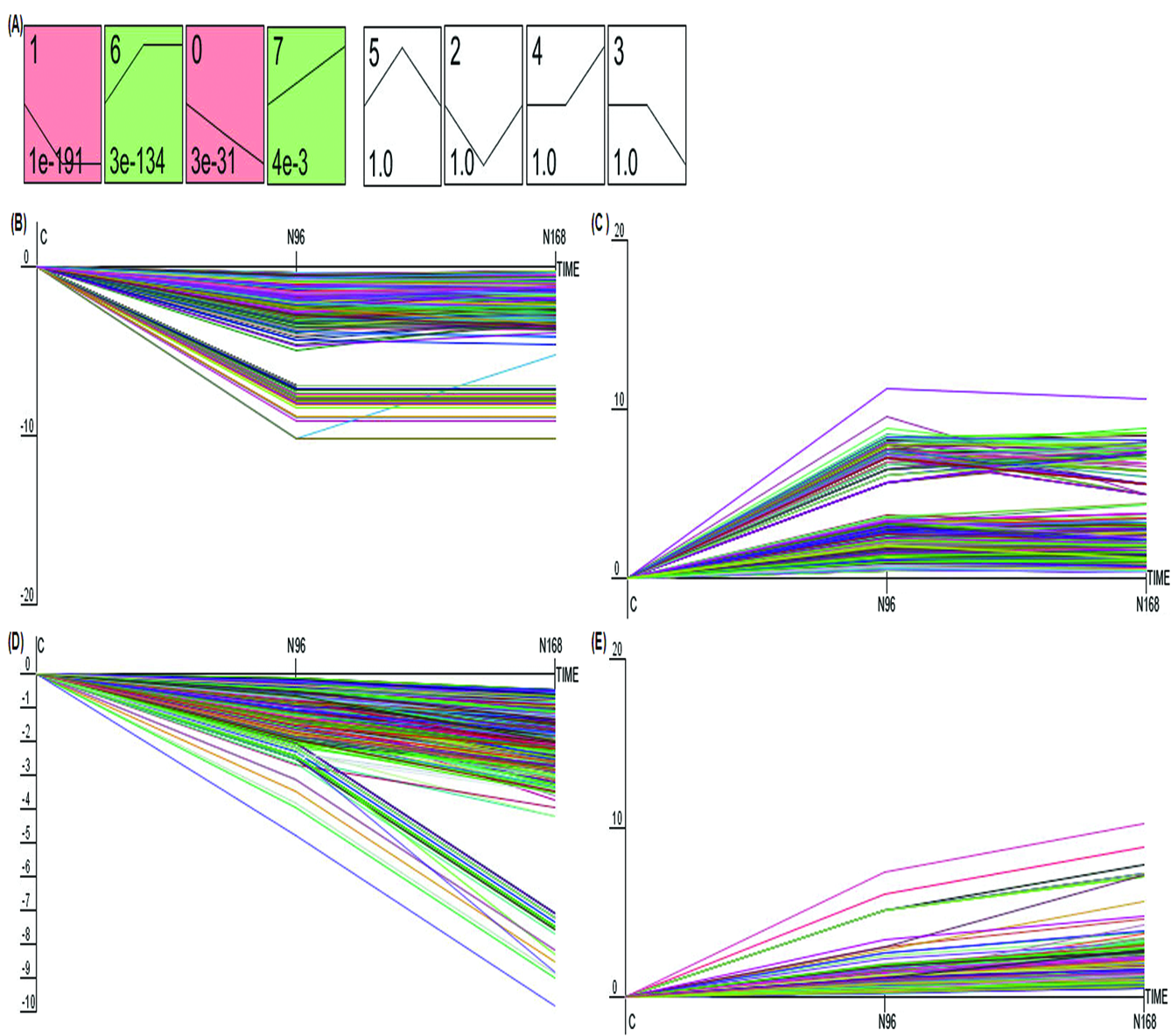

Supplement: Figure S6 — STC (Series Test of Cluster) analysis of DE genes. Dynamic gene expression profiles in all 5430 DE genes are shown for eight clusters (A). The eight profiles were ordered based on the p value significance of number of genes assigned versus expected. The upper left represents the serial number of the cluster, and the under left represents the p value. (B–E) four significant cluster profiles which have significantly more genes assigned under the true ordering of time points compared to the average number assigned to the model profile in the permutation runs. Y axis indicates the relative gene expression change presented in log2 ratio between UNC lung and N-PRRSV infected lungs at the indicated time points. (B) profile 1 (0,−1,−1), 595.3 genes were expected, but 1373.0 genes were assigned, p-value = 1.0E-191; (C) profile 6 (0,1,1), 810.7 genes were expected, but 1520.0 genes were assigned, p-value = 3.0E-134; (D) profile 0 (0,−1,−2), 646.0 genes were expected, but 404.0 genes were assigned, p-value = 2.8E-31; (E) profile 7 (0,1,2), 456.0 genes were expected, but 404.0 genes were assigned, p-value = 4.4E-3. (3.73 MB TIF) [file pone.0011377.s006.tif]

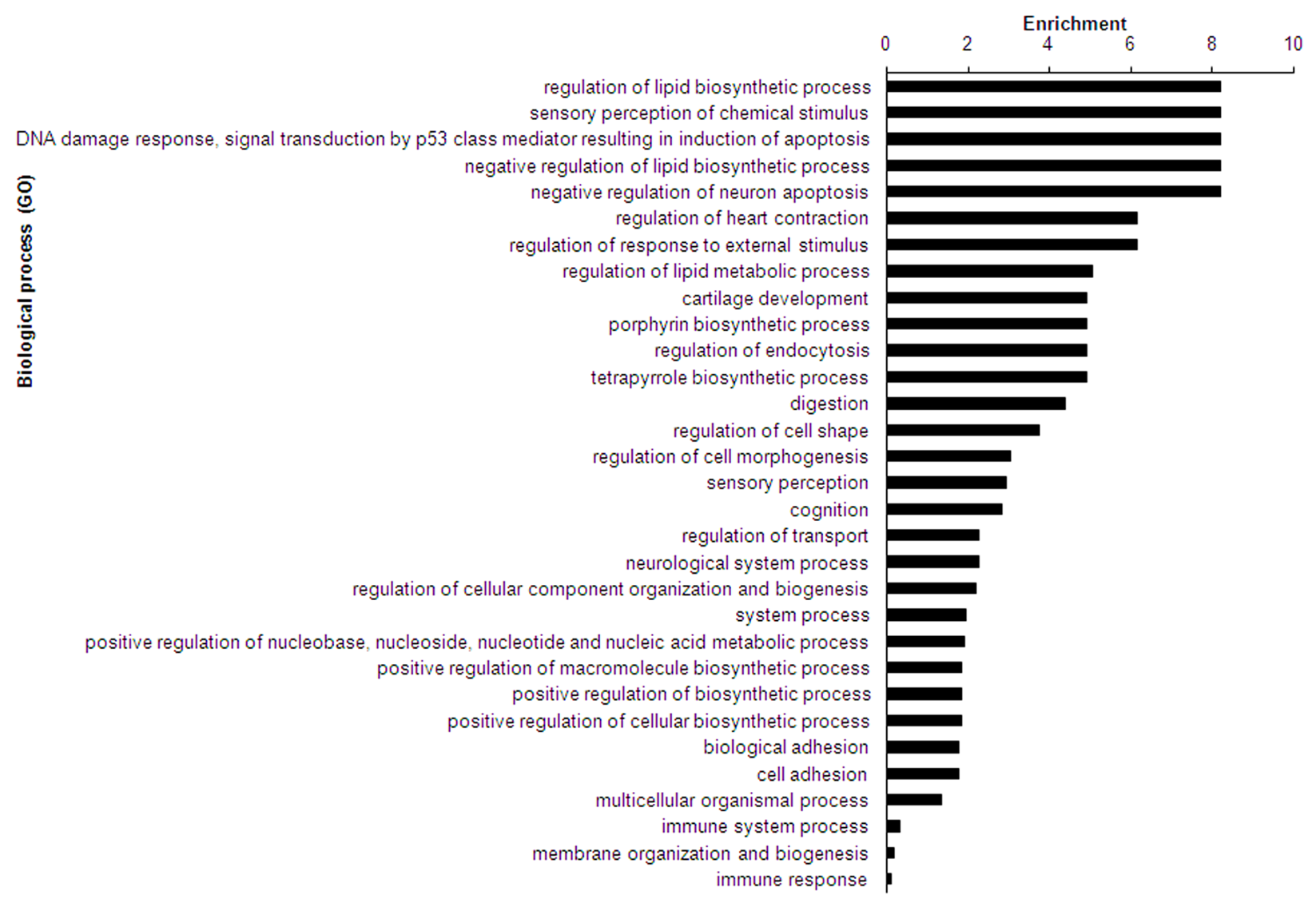

Supplement: Figure S7 — Biological process GO terms of profile 1. Functional classification of the DE genes was performed according to GO biological processes. A P-value of <0.05 in the two-side Fisher's exact test were selected as the significant criteria. These DE genes were sorted by the enrichment of GO categories. The vertical axis is the GO category and the horizontal axis is the enrichment of GO. (1.53 MB TIF) [file pone.0011377.s007.tif]

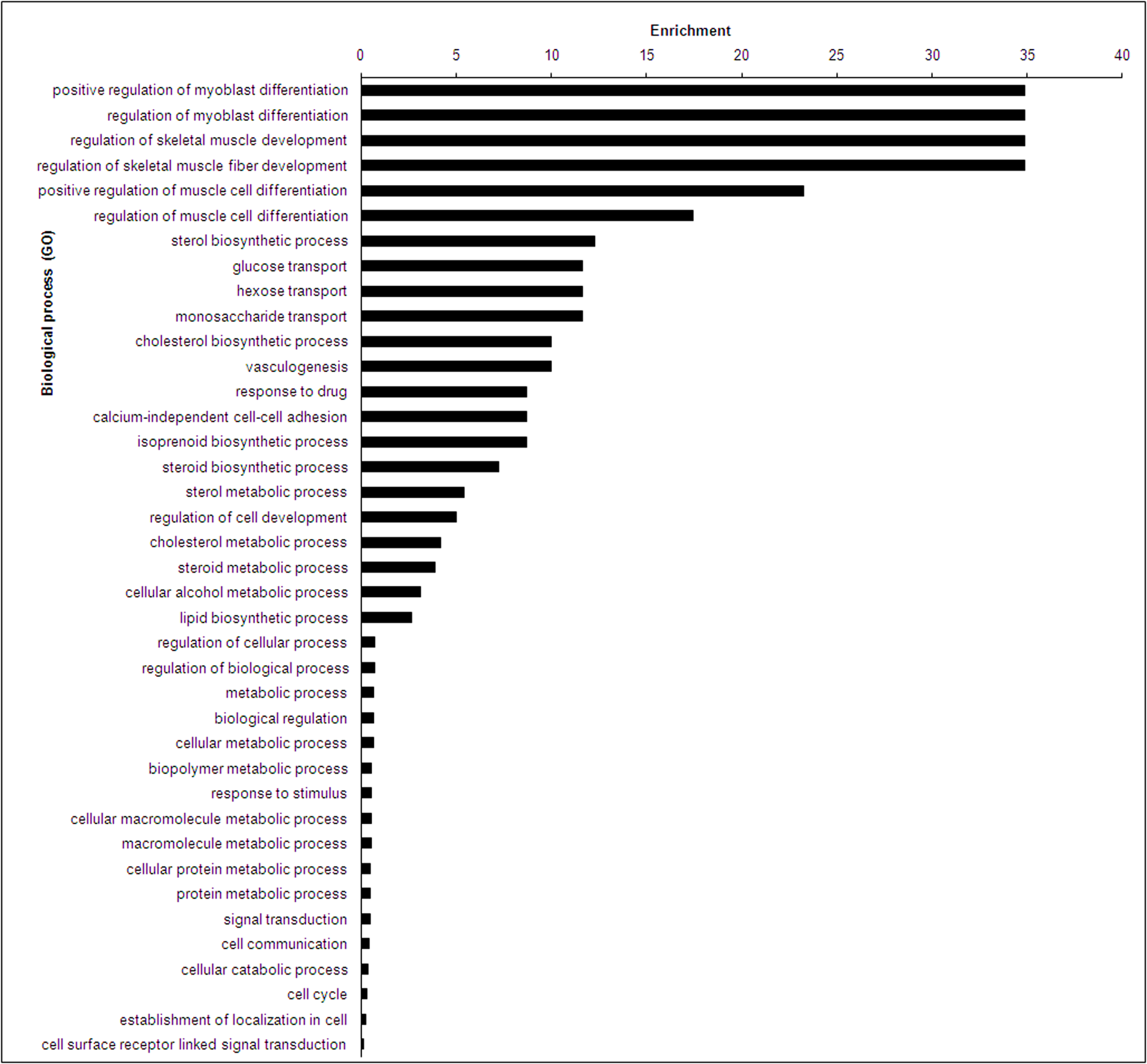

Supplement: Figure S8 — Biological process GO terms of profile 0. Functional classification of the DE genes was performed according to GO biological processes. A P-value of <0.05 in the two-side Fisher's exact test were selected as the significant criteria. These DE genes were sorted by the enrichment of GO categories. The vertical axis is the GO category and the horizontal axis is the enrichment of GO. (2.78 MB TIF) [file pone.0011377.s008.tif]

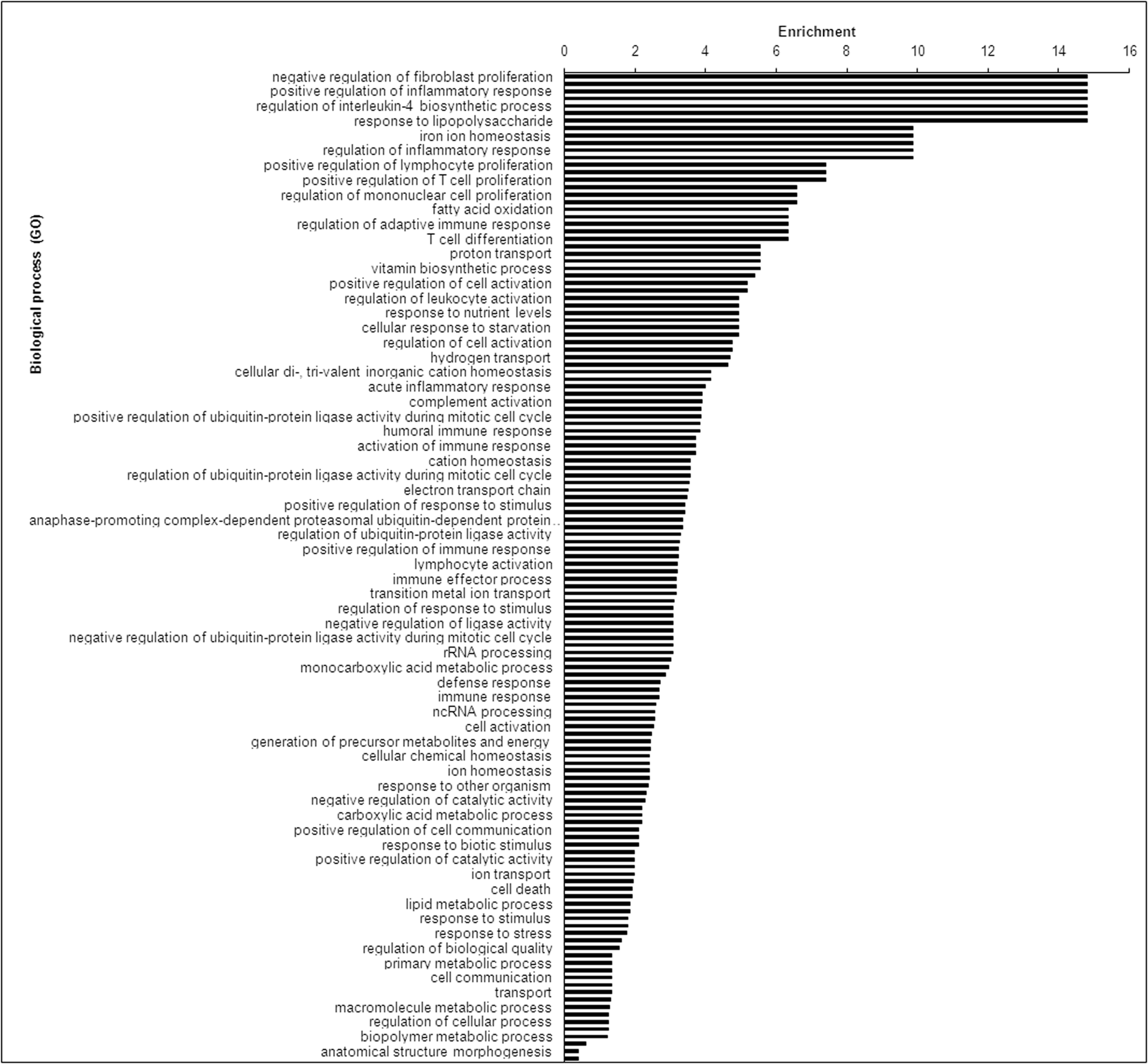

Supplement: Figure S9 — Biological process GO terms of profile 6. Functional classification of the DE genes was performed according to GO biological processes. A P-value of <0.05 in the two-side Fisher's exact test were selected as the significant criteria. These DE genes were sorted by the enrichment of GO categories. The vertical axis is the GO category and the horizontal axis is the enrichment of GO. (1.44 MB TIF) [file pone.0011377.s009.tif]

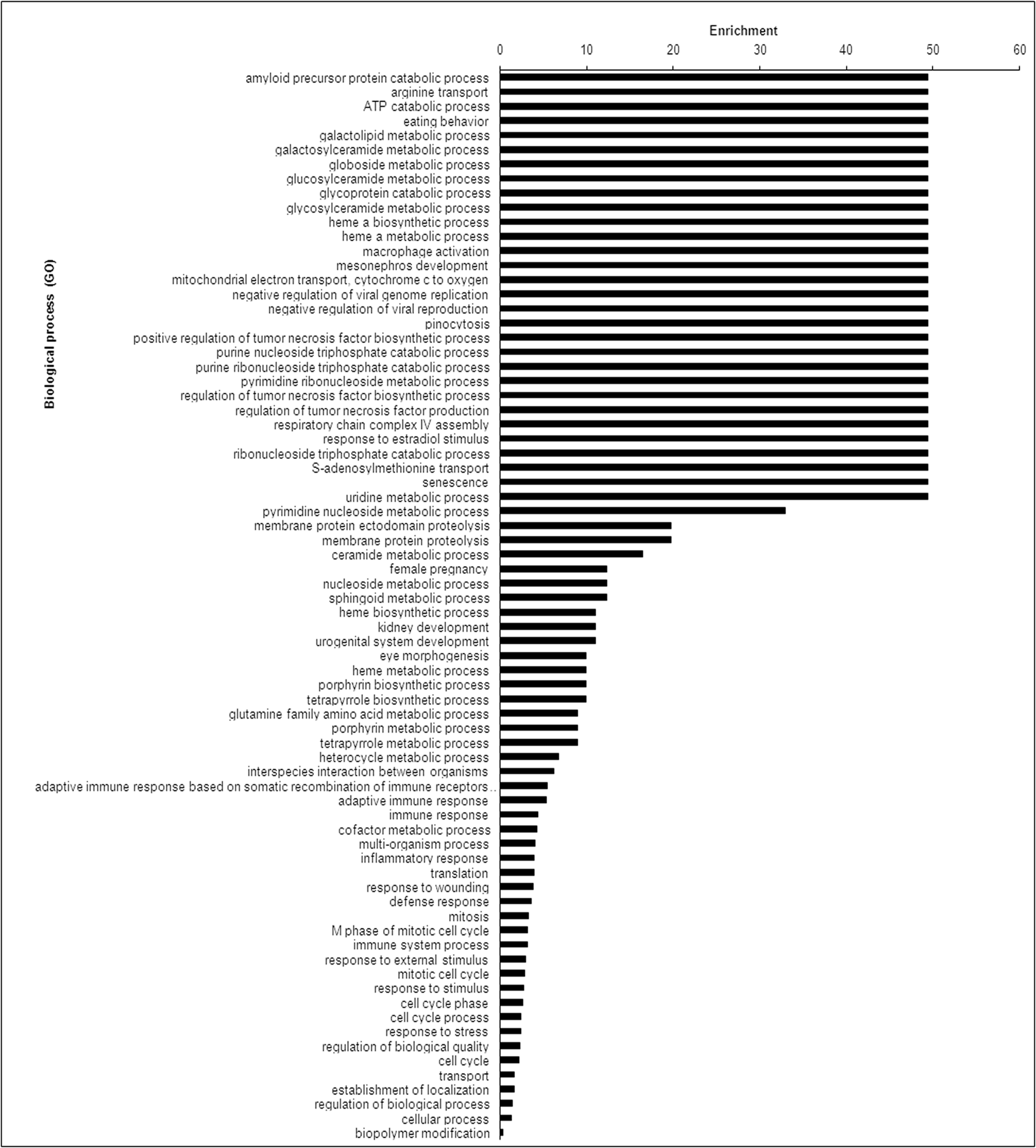

Supplement: Figure S10 — Biological process GO terms of profile 7. Functional classification of the DE genes was performed according to GO biological processes. A P-value of <0.05 in the two-side Fisher's exact test were selected as the significant criteria. These DE genes were sorted by the enrichment of GO categories. The vertical axis is the GO category and the horizontal axis is the enrichment of GO. (1.56 MB TIF) [file pone.0011377.s010.tif]
