# Supplementary material for: Understanding PRRSV Infection in Porcine Lung Based on Genome-Wide Transcriptome Response Identified by Deep Sequencing
Source: PLoS One. 2010 Jun 29;5(6):e11377. doi: 10.1371/journal.pone.0011377 (PMC2894071; doi:10.1371/journal.pone.0011377)
Supplement: Table S1 — QPCR results of N-PRRSV NSP2 gene expression in infected pigs. (0.03 MB DOC) [file pone.0011377.s011.doc]

| **Groups** | **Samples** | **Mean** | **SD** |
| --- | --- | --- | --- |
|  | 4 | 31.1746 | 0.65428 |
|  | 5 | 33.4095 | 1.55387 |
|  | 6 | 33.2089 | 0.31566 |
| N96 |  | 32.5977 | 1.34594 |
|  | 7 | 33.1597 | 0.82849 |
|  | 8 | 34.1037 | 1.22252 |
|  | 9 | 31.8236 | 1.29257 |
| N168 |  | 33.029 | 1.32379 |

Mean: Mean of Cycle threshold value.

SD: Standard deviation.
